# Bioactivity research in *Grifola frondosa*: a global scientometric analysis of emerging bioactivities and translational applications

**DOI:** 10.3389/ffunb.2026.1940029

**Published:** 2026-10-07

**Authors:** Christian Joseph N. Ong, Jamil Allen G. Fortaleza, Kevin Smith P. Cabuhat, Glocielle F. Molabola, Johnalie N. Cabalbag, Jose Jurel M. Nuevo, Jowi Tsidkenu Pili Cruz, Esperanza C. Cabrera

**Affiliations:** 1 Department of Biology, College of Science, De La Salle University, Manila, Philippines; 2 Department of Biology, College of Arts and Sciences, Manila Central University, Caloocan, Philippines; 3 National University, Manila, Philippines; 4 Research Fellow, Bulacan State University, Malolos, Bulacan, Philippines; 5 Basic Education Department, La Consolacion University Philippines, Malolos, Bulacan, Philippines; 6 Molecular Science Unit Laboratory, Center for Natural Sciences and Environmental Research, De La Salle University, Manila, Philippines; 7 College of Medical Laboratory Sciences, Our Lady of Fatima University, Valenzuela, Philippines

**Keywords:** bioactivity, *Grifola frondosa*, medicinal mushrooms, nutraceuticals, research trends, scientometric analysis, β-glucans

## Abstract

**Introduction:**

*Grifola frondosa* (maitake) is an edible and medicinal mushroom known for its diverse bioactive compounds, particularly polysaccharides, including biologically active β-glucans, which have been associated with immunomodulatory, antioxidant, antitumor, antimicrobial, and metabolic effects. However, a comprehensive assessment of the intellectual structure and global development of this research area has not yet been conducted.

**Methods:**

We conducted a scientometric analysis of 605 English-language journal articles indexed in Scopus from 1980 to 2025. Bibliometric data were analyzed using Bibliometrix/Biblioshiny and VOSviewer to assess publication and citation trends, leading authors, institutions, countries, collaboration networks, keyword co-occurrence, thematic evolution, conceptual structure, and emerging research frontiers.

**Results:**

Publication output rose significantly after 2024, peaking in 2025, reflecting sustained global interest in *G. frondosa* bioactivity. Logistic growth modeling shows the field is nearing maturity. China and Japan showed the highest publication output and substantial representation in institutional and international collaboration networks, indicating a pronounced concentration of the analyzed literature in East Asia. Keyword and thematic analyses highlight β-glucans, polysaccharides, immunomodulation, antioxidant activity, and antitumor mechanisms as core research themes. Recent studies increasingly address structural characterization, gut microbiota modulation, fermentation technologies, oxidative stress, hypoglycemic activity, and functional food applications, indicating a shift toward mechanistic and translational research.

**Discussion:**

Research on *G. frondosa* bioactivity has evolved into a multidisciplinary field characterized by expanding international collaboration and heightened focus on translational research. The identified trends, knowledge structure, and emerging themes offer a framework for future mechanistic, preclinical, and clinical studies exploring the therapeutic, nutraceutical, and functional food applications of *G. frondosa*.

## 1 Introduction

*Grifola frondosa*, commonly known as maitake or hen-of-the-woods, is an edible and medicinal mushroom that has attracted considerable scientific interest because of its nutritional value, traditional use, and diverse biological activities. Its fruiting bodies and mycelia contain a chemically heterogeneous array of bioactive constituents, including polysaccharides (notably β-glucans), proteins and glycoproteins, phenolic compounds, terpenoids, sterols, and other low-molecular-weight metabolites that may contribute individually or synergistically to its functional properties (Camilleri et al., 2024). Correspondingly, *G. frondosa* has been investigated for a broad range of biological and pharmacological activities, including immunomodulatory, antitumor, antioxidant, anti-inflammatory, hypoglycemic, antimicrobial, antiviral, and metabolic effects (Wu et al., 2021; He et al., 2018). This chemical and functional diversity has positioned *G. frondosa* at the intersection of medicinal mushroom research, functional food development, natural-product pharmacology, and preventive health research.

The bioactivity landscape of *G. frondosa* encompasses multiple compounds, mechanisms, and biological targets. Polysaccharides, including β-glucan-rich fractions such as D-fraction and grifolan, constitute one of the historically prominent areas of investigation and have been associated particularly with immunomodulatory and antitumor activities. Experimental evidence indicates that these macromolecules can influence macrophages, dendritic cells, natural killer cells, and T lymphocytes and interact with pattern-recognition receptors, including Dectin-1 and complement receptor 3, thereby modulating cytokine production and innate and adaptive immune responses (Vetvicka and Vetvickova, 2014; Murphy et al., 2020). However, the biological potential of *G. frondosa* extends beyond polysaccharide-mediated effects. Proteins and glycoproteins, phenolic constituents, sterols, terpenoid-related compounds, and other metabolites have also been investigated for their contributions to antioxidant, antimicrobial, metabolic, and other biological activities. Accordingly, the bioactivity of *G. frondosa* is more appropriately considered as arising from a chemically diverse repertoire of constituents rather than being attributed exclusively to a single compound or compound class.

Beyond immunomodulation and antitumor activity, research has increasingly examined the antioxidant, anti-inflammatory, antidiabetic, antiviral, neuroprotective, and metabolic properties of *G. frondosa*. Extracts, fractions, and isolated constituents have demonstrated free-radical-scavenging activity and have been investigated for their effects on glucose homeostasis, lipid metabolism, microbial growth, viral processes, inflammatory responses, and other physiological pathways (Yeh et al., 2011). Advances in chemical characterization and metabolomic approaches have further expanded attention toward phenolic compounds and other metabolites that may contribute to these effects. More recently, studies examining interactions between *G. frondosa*-derived constituents and the gut microbiota, host metabolism, and immune regulation have broadened the mechanistic landscape of the field (Zhong et al., 2023; Sung et al., 2023; Camilleri et al., 2024). These developments illustrate a progressive shift from investigating individual biological effects toward understanding interconnected molecular, microbial, metabolic, and physiological mechanisms underlying the functional properties of the mushroom.

With growing interest in the therapeutic potential of *G. frondosa*, the number of scientific publications addressing its bioactivity has increased substantially. Research on this topic now encompasses diverse disciplines, including pharmacology, immunology, biotechnology, food science, oncology, and nutraceutical development. Previous reviews have synthesized evidence on the bioactive constituents of edible and medicinal mushrooms, including polysaccharides (particularly β-glucans), proteins, phenolic compounds, sterols, and terpenoids, as well as their immunomodulatory, antioxidant, antitumor, metabolic, hepatoprotective, and other health-related activities (Adesida and Alimba, 2025; Adesida et al., 2026). However, these reviews primarily synthesize the composition, biological activities, health effects, and translational potential of mushroom-derived bioactive compounds rather than quantitatively examining the structure and evolution of the scientific literature specifically on *G. frondosa*. Consequently, a comprehensive scientometric perspective on the development of *G. frondosa* bioactivity research, including its temporal growth, geographic and institutional distribution, collaboration networks, intellectual structure, thematic evolution, and emerging research fronts remains limited. Scientometric analysis provides a complementary framework for examining these dimensions by quantitatively characterizing publication and citation patterns, intellectual and collaborative structures, and the temporal evolution of research topics. Such an approach is particularly relevant to *G. frondosa* because its literature encompasses multiple bioactive compound classes and biological effects rather than a single therapeutic constituent or mechanism.

Therefore, a comprehensive scientometric evaluation of bioactivity research on *G. frondosa* is warranted to characterize the field as a whole rather than emphasizing any individual bioactive compound or pharmacological effect. This study analyzes global publication and citation trends; identifies leading authors, institutions, countries, and journals; evaluates patterns of scientific collaboration; maps the conceptual and thematic structure of the literature; examines the evolution of major research themes; and identifies emerging areas of investigation. By integrating these dimensions, the study provides a global perspective on how research concerning the diverse bioactive constituents and biological properties of *G. frondosa* has developed and may inform future mechanistic, clinical, nutraceutical, and functional food research.

## 2 Methods

### 2.1 Study design

The study employed scientometric approaches to characterize the structural evolution of bioactivity research in *G. frondosa*. Scientometrics enables the quantitative assessment of scientific productivity, collaborative networks, and thematic trajectories through analysis of bibliographic metadata, co-authorship linkages, and keyword co-occurrence patterns. This approach offers a systematic means to trace the growth, diversification, and emerging directions of a research field.

### 2.2 Data source

Scopus was chosen as the main data source due to its broad coverage of fungal biology and applied mycology journals, strong citation tracking, and frequent use in scientometric studies. Compared with Web of Science, Scopus covers more applied mycology and fungal biology journals, making it well-suited for mapping trends in bioactivity research in *G. frondosa*. Using Scopus alone keeps metadata uniform and greatly reduces duplicate records. While using more databases can slightly increase coverage, it often results in inconsistent citation data and metadata, particularly in author names, institutional affiliations, and keyword use. These inconsistencies require much extra cleanup and can introduce bias through matching errors or split citation counts. Sticking to a single comprehensive database keeps bibliometric fields such as authorship, affiliations, and citation counts consistent and standardized, which is key to reliable network analysis and reproducible results. This approach matches accepted scientometric practices, which focus on structural mapping rather than the exhaustive approach of systematic reviews. Scopus also allows detailed metadata export, including subject classifications and CiteScore indicators, to help contextualize influential sources. The extracted metadata included publication year, authorship, institutional affiliations, journal sources, keywords, abstracts, subject categories, and citation counts. However, relying on a single database can underrepresent locally published or unindexed work from lower-income regions. Any observed differences in research output should thus be seen as differences in indexed visibility, not absolute research activity.

### 2.3 Search strategy and data extraction

A structured search strategy was developed to maximize retrieval sensitivity while maintaining conceptual specificity and global relevance. Data retrieval was conducted in Scopus on June 17, 2026, using the TITLE-ABS-KEY fields to capture publications in which validated keywords related to bioactivity research in *G. frondosa* appeared in article titles, abstracts, or author/index keywords. Although the search was conducted in 2026, the analytical period was intentionally restricted to publications indexed through December 2025. Records published in 2026 were excluded because the year was incomplete at the time of data retrieval and their inclusion would have represented only a partial publication year. Such partial-year data could artificially underestimate annual scientific production and introduce temporal bias into longitudinal analyses of publication growth, citation patterns, and thematic development. Restricting the dataset to complete calendar years therefore ensured temporal comparability across the study period and provided a consistent endpoint for the scientometric analyses. To improve transparency and reproducibility in bibliographic retrieval and screening, the study-selection workflow was adapted from PRISMA-style reporting principles, encompassing record identification, screening, eligibility assessment, and final inclusion of publications for scientometric analysis (**Figure 1**).

**Figure 1:**
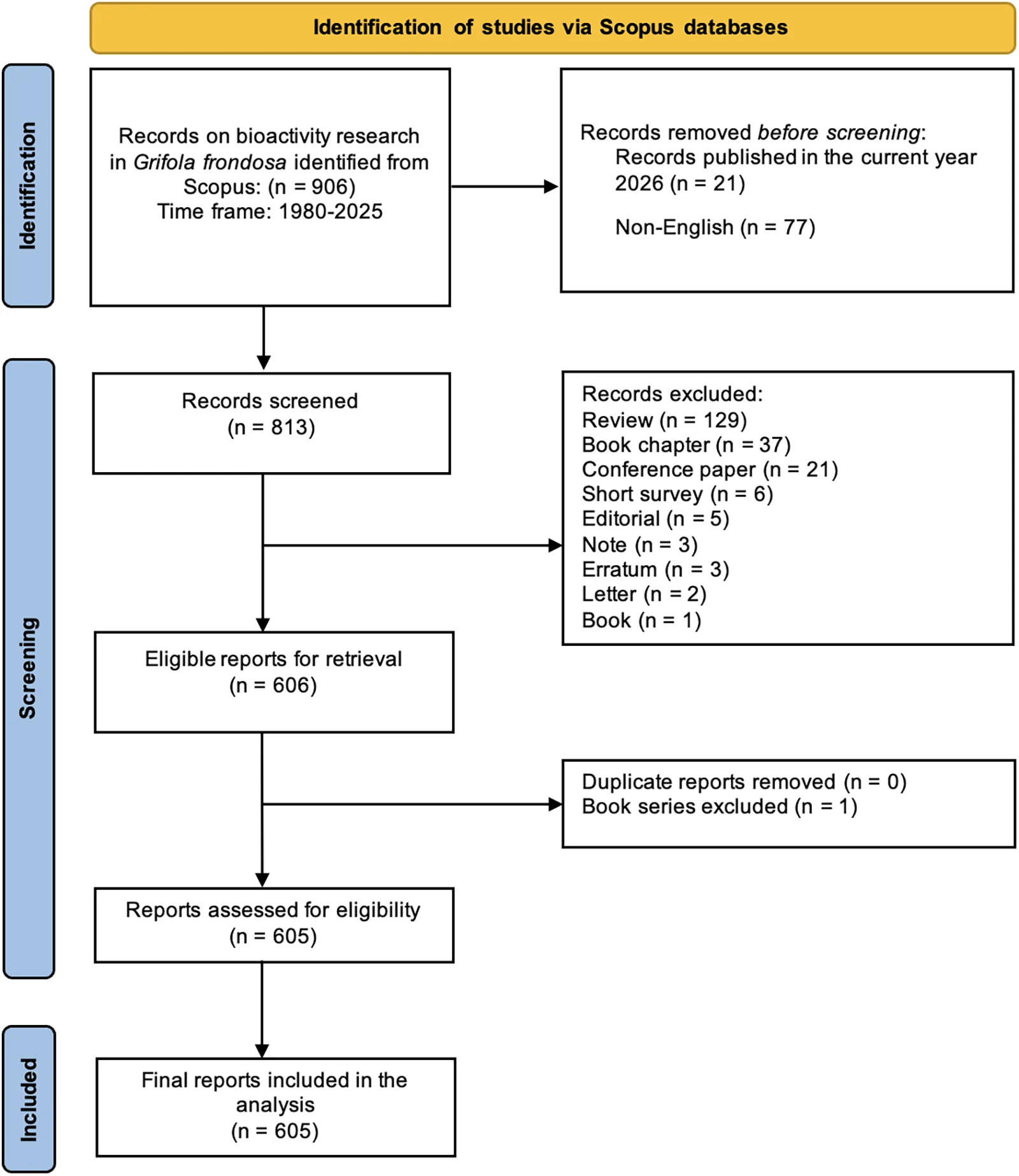
PRISMA-inspired flow diagram of the scientometric study on bioactivity research in *G. frondosa*.

The analysis was restricted to original research articles published in English. Original articles were selected because they represent primary contributions to the scientific literature and provide an appropriate basis for evaluating the generation and development of empirical knowledge, including publication productivity, citation patterns, collaboration networks, and the evolution of research themes. Review articles, systematic reviews, meta-analyses, conference papers, book chapters, editorials, and other non-original publication types were excluded because they primarily synthesize, reinterpret, or summarize previously published findings rather than report independent primary research. In particular, including review articles could disproportionately amplify already established topics, highly cited studies, authors, and research areas through secondary synthesis, potentially affecting the interpretation of productivity, citation, and thematic patterns. Restricting the dataset to original research articles therefore provided a more methodologically homogeneous corpus for characterizing the development and intellectual structure of primary bioactivity research on *G. frondosa*. This exclusion should not be interpreted as diminishing the scientific importance of review literature but rather as a methodological decision aligned with the specific objectives and unit of analysis of the present scientometric study.

The first search block incorporated the accepted scientific name (*“Grifola frondosa”*) together with its widely recognized common names (“Maitake” and “Maitake mushroom”). Combining scientific and common nomenclature improved retrieval sensitivity because terminology may vary according to disciplinary convention, geographic context, and author preference, thereby reducing the likelihood of excluding relevant publications because of nomenclatural variation. The second search block encompassed a broad spectrum of biological activities, pharmacological effects, and bioactive constituents associated with *G. frondosa*. Activity-related terms included antioxidant, anti-inflammatory, anticancer, antitumor, cytotoxic, immunomodulatory, immunostimulatory, antimicrobial, antibacterial, antiviral, antidiabetic, hepatoprotective, neuroprotective, and cardioprotective effects, representing major areas investigated in the bioactivity literature. Broader descriptors, including pharmacological, therapeutic, and medicinal, were incorporated to retrieve studies addressing biological or health-related activities that might not be indexed using a specific activity term.

To broaden the retrieval of mechanistic, biochemical, and compositional studies, the search strategy additionally incorporated major classes of bioactive constituents associated with *G. frondosa*, including polysaccharides, β-glucans, glycoproteins, phenolics, polyphenols, sterols, and the broader descriptor “bioactive compounds.” These constituents have been widely investigated in relation to the biological and functional properties of *G. frondosa* and therefore represent important descriptors for capturing studies that may emphasize compound characterization rather than a specific pharmacological activity in their titles, abstracts, or keywords. Wildcard operators (*) were applied where appropriate to capture plural forms and related lexical variants, thereby improving search sensitivity while maintaining conceptual specificity. Both Greek-letter (“β-glucan”) and text-based (“beta-glucan”) variants were incorporated to account for differences in author terminology and database indexing. Collectively, the combination of organism-specific terminology, activity-related descriptors, and bioactive-compound terms was designed to achieve a balance between retrieval sensitivity and specificity while maintaining a clearly defined corpus of primary research relevant to the scientometric objectives of the study.

The Scopus advanced search query was designed to capture scholarly articles related to bioactivity research in *G. frondosa* and was applied to article titles and abstracts. The full search string used in this study is presented below:

TITLE-ABS-KEY (“Grifola frondosa” OR Maitake OR “Maitake mushroom”) AND TITLE-ABS-KEY (antioxidant OR anti-inflammatory OR anticancer OR antitumor OR cytotoxic OR immunomodulatory OR immunostimulatory OR antimicrobial OR antibacterial OR antiviral OR antidiabetic OR hepatoprotective OR neuroprotective OR cardioprotective OR pharmacological OR therapeutic OR medicinal OR polysaccharide* OR “beta-glucan*” OR β-glucan* OR glycoprotein* OR phenolic* OR polyphenol* OR sterol* OR “bioactive compound*”) AND (LIMIT-TO (SRCTYPE, “j”)) AND (LIMIT-TO (DOCTYPE, “ar”)) AND (EXCLUDE (PUBYEAR, “2026”)) AND (LIMIT-TO (LANGUAGE, “English”))

### 2.4 Data cleaning and harmonization

Manual data cleaning and bibliographic standardization were performed to improve the accuracy and consistency of the bibliometric dataset. The process included removing duplicate records, standardizing author and institutional names, and harmonizing keywords using a manually curated thesaurus file. Author and institutional name variations were systematically unified to reduce fragmentation in co-authorship and collaboration networks. Minimum inclusion thresholds were applied to improve the interpretability and analytical stability of the bibliometric networks by limiting the influence of highly infrequent terms and entities. For keyword co-occurrence analysis, a threshold of at least five occurrences was selected to retain recurrent concepts that demonstrated sufficient representation across the 605-article corpus while excluding isolated or very low-frequency terms that could increase network sparsity and visual clutter without substantially contributing to the identification of the field’s major conceptual structures. For institutional analysis, a minimum threshold of ten publications was applied to focus the network and productivity assessment on institutions demonstrating sustained contributions to the literature rather than those represented by only sporadic publications. These thresholds were therefore used as pragmatic, dataset-specific filtering criteria to balance inclusiveness with network readability and meaningful structural interpretation; they should not be interpreted as universal cutoffs for scientometric analyses.

Keyword normalization subsequently merged synonymous terms, spelling variants, and indexing inconsistencies. For example, “*Grifola frondosa*,” “maitake,” and “maitake mushroom” were standardized under one descriptor, while related terms such as “β-glucan,” “β-glucans,” “β-1,3-glucan,” and “grifolan” were harmonized. Bioactivity-related terms, including “antioxidant activity,” “antitumor activity,” and “immunomodulatory activity,” were likewise consolidated. Non-informative terms, spelling errors, and indexing artifacts were removed to reduce analytical noise and improve the reliability and interpretability of thematic mapping and network analyses. Following database retrieval and application of the predefined eligibility criteria, all remaining records were independently screened by two authors based on their titles and abstracts to determine their relevance to bioactivity research on *G. frondosa*. Studies retrieved through incidental keyword overlap but not directly addressing the biological activities, bioactive constituents, or related functional properties of *G. frondosa* were excluded. Any discrepancies in eligibility assessments between the two reviewers were resolved through discussion and consensus; when the relevance of a record could not be determined from the title and abstract alone, the full text was consulted to establish eligibility. This independent screening procedure was undertaken to minimize subjective selection bias and false-positive inclusion, reduce database noise, and enhance the consistency and reproducibility of the final scientometric dataset.

### 2.5 Data analysis

This bibliometric study used science mapping and performance analysis on the retrieved dataset (.csv) with Bibliometrix/Biblioshiny (R package) and VOSviewer version 1.6.20 (Universiteit Leiden and CWTS Meaningful Metrics). These tools supported the visualization of network maps, thematic diagrams, trend figures, and conceptual structure mapping through strategic thematic maps, thematic evolution, and multidimensional scaling (MDS). Bibliometrix calculated publication and citation metrics, such as annual publication counts, top journals, trend topics, and leading institutions (Aria and Cuccurullo, 2017). VOSviewer mapped co-authorship and co-occurrence networks to analyze inter-country and inter-institutional collaborations, as well as keyword centrality structures (van Eck and Waltman, 2010). Thematic evolution was assessed through keyword co-occurrence analysis of titles and abstracts, generating cluster maps and temporal overlays to identify dominant research areas and emerging topics. **Figure 2** presents a schematic workflow of the scientometric methodology, including database retrieval, search strategy, selection process, data export, enrichment analysis with Bibliometrix/Biblioshiny and VOSviewer, and subsequent science mapping and interpretation.

**Figure 2:**
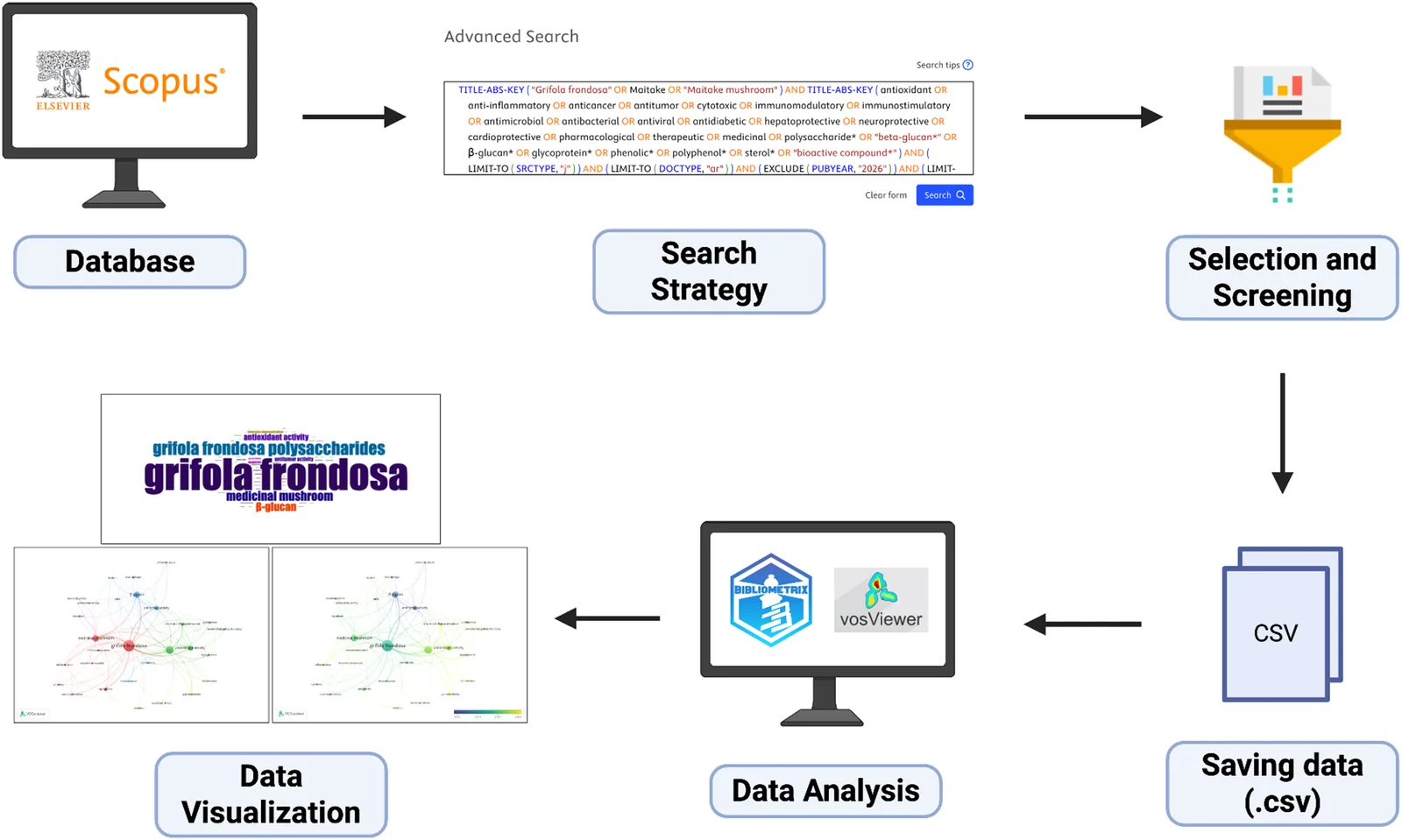
Schematic workflow of the scientometric analysis conducted in this study, illustrating the processes of database retrieval from Scopus, search strategy formulation, study selection and data exportation, followed by enrichment and science mapping analyses using Bibliometrix/Biblioshiny (R package) and VOSviewer for visualization.

A focused narrative synthesis of the underlying literature was incorporated into the Discussion to contextualize the biological relevance of the dominant themes identified through scientometric analyses. This synthesis explains the pharmacological and mechanistic context of the identified research themes and serves as a complement to, rather than an extension of, the bibliometric evidence.

## 3 Results

### 3.1 Annual publication, citation trends and temporal dynamics

A total of 605 original articles were retrieved and analyzed from the Scopus database. The annual publication trend indicates that bioactivity research on *G. frondosa* developed slowly during the early period, with only sporadic outputs from the 1980s to the late 1990s (**Figure 3**). Publication activity remained low and fluctuating, suggesting that early investigations were limited to isolated studies on its medicinal, nutritional, or polysaccharide-related properties. A visible increase began in the early 2000s, followed by stronger growth after 2005, reflecting expanding interest in *G. frondosa* as a bioactive medicinal mushroom.

**Figure 3:**
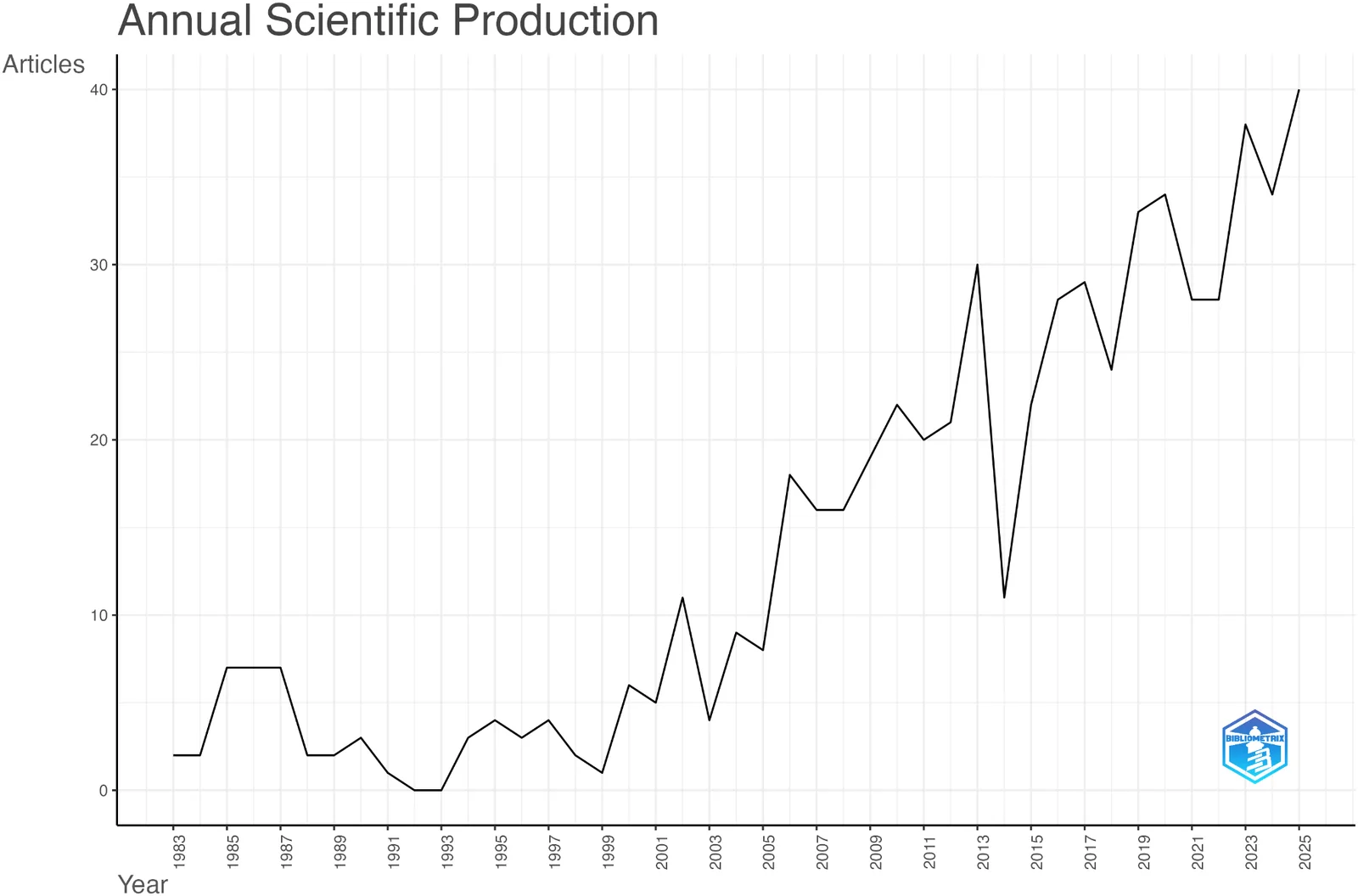
Trend analysis of annual scientific publications on bioactivity research in *G. frondosa* (1983-2025).

From the mid-2010s onward, the field entered a more active and sustained growth phase, with annual publications generally exceeding 20 articles and reaching the highest output in 2025 at approximately 40 articles. Although intermittent declines are observed, particularly around 2014 and 2021–2022, the overall trajectory remains upward. This pattern suggests increasing scientific attention to *G. frondosa* bioactivities, likely driven by research on polysaccharides, β-glucans, immunomodulatory effects, antioxidant activity, antitumor potential, and functional food or nutraceutical applications.

The citation trend shows a fluctuating but progressively strengthening impact of *G. frondosa* bioactivity research (**Figure 4**). Early citation averages from the 1980s to late 1990s remained relatively modest and irregular, reflecting a small and emerging literature base. Citation activity increased more visibly in the early 2000s, with notable peaks around 2002–2004, suggesting that studies published during this period gained stronger scholarly attention, likely due to growing interest in maitake polysaccharides, β-glucans, and immunomodulatory or antitumor properties.

**Figure 4:**
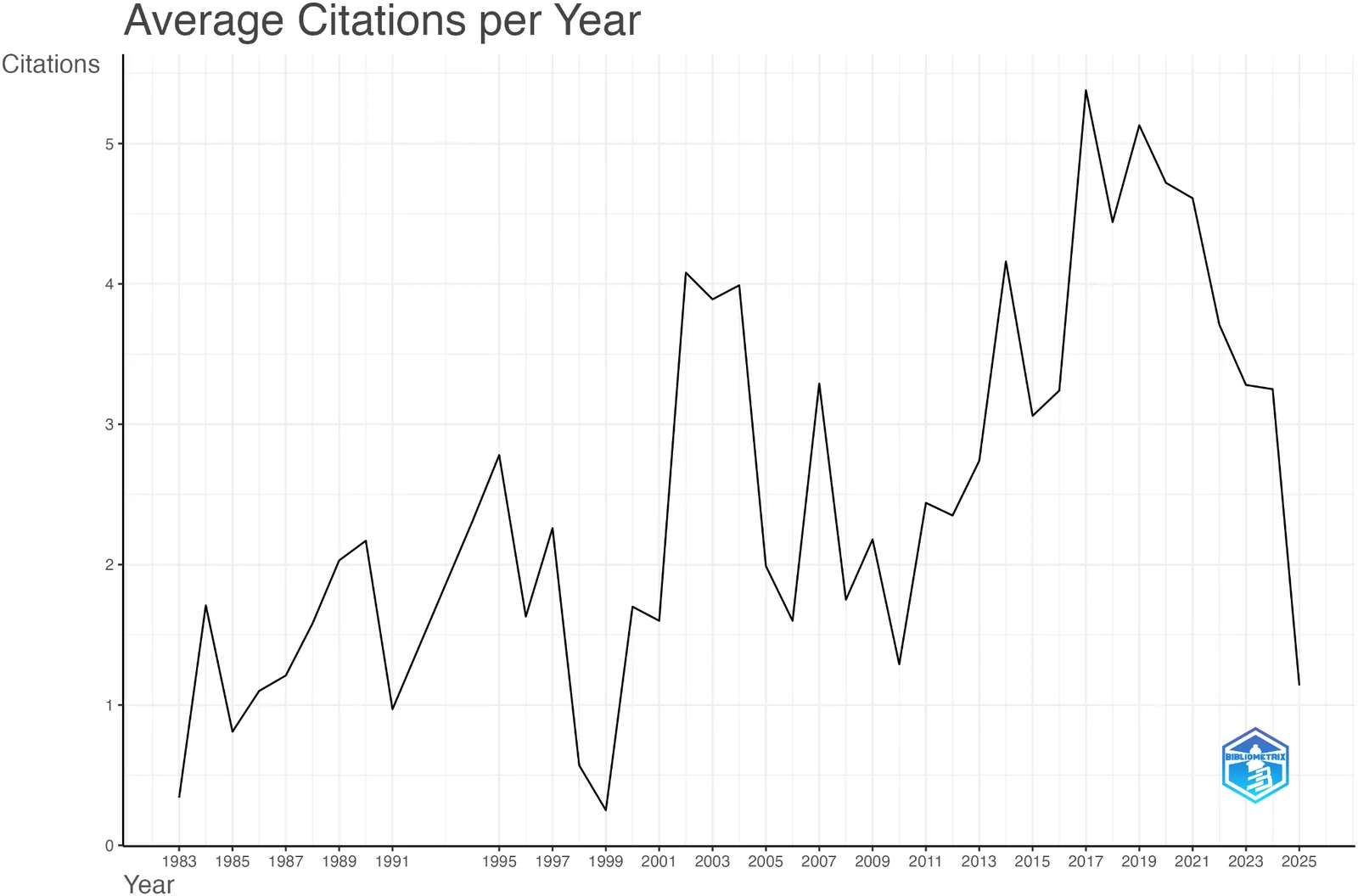
Citation trend analysis of bioactivity research in *G. frondosa* (1983-2025).

Citation impact became more pronounced from the mid-2010s onward, with the highest average citation level observed around 2017, followed by sustained high values through 2019–2021. This pattern suggests that recent studies on the antioxidant, immunomodulatory, antitumor, and functional food-related properties of *G. frondosa* have gained greater visibility within natural products and medicinal mushroom research. The apparent decline after 2022, particularly in 2025, should be interpreted with caution because newer publications have had less time to accumulate citations, making recent citation averages sensitive to citation-window effects.

The life cycle analysis shows that annual publications on *G. frondosa* bioactivity research followed a logistic life-cycle pattern, with slow early development from the 1980s to the late 1990s, followed by accelerated growth after the early 2000s (**Figure 5A**). The observed publication points show a clear expansion phase from approximately 2005 onward, reaching the highest annual output in the 2020s. The fitted model shows strong explanatory performance (R² = 0.895) and projects a peak of approximately 37 publications around 2027.7, suggesting that the field is approaching a mature phase after several decades of progressive expansion.

**Figure 5B** further supports this maturation pattern, showing cumulative publications increasing slowly during the early period before entering a steep growth phase after 2010. The logistic model estimates a saturation level of approximately 1,403 cumulative publications, with the curve indicating that the field has not yet reached its projected upper limit. The rapid rise toward the 50% threshold reflects intensifying scholarly attention to *G. frondosa* bioactivity, particularly in relation to polysaccharides, β-glucans, antioxidant, immunomodulatory, antitumor, and functional food applications.

**Figure 5:**
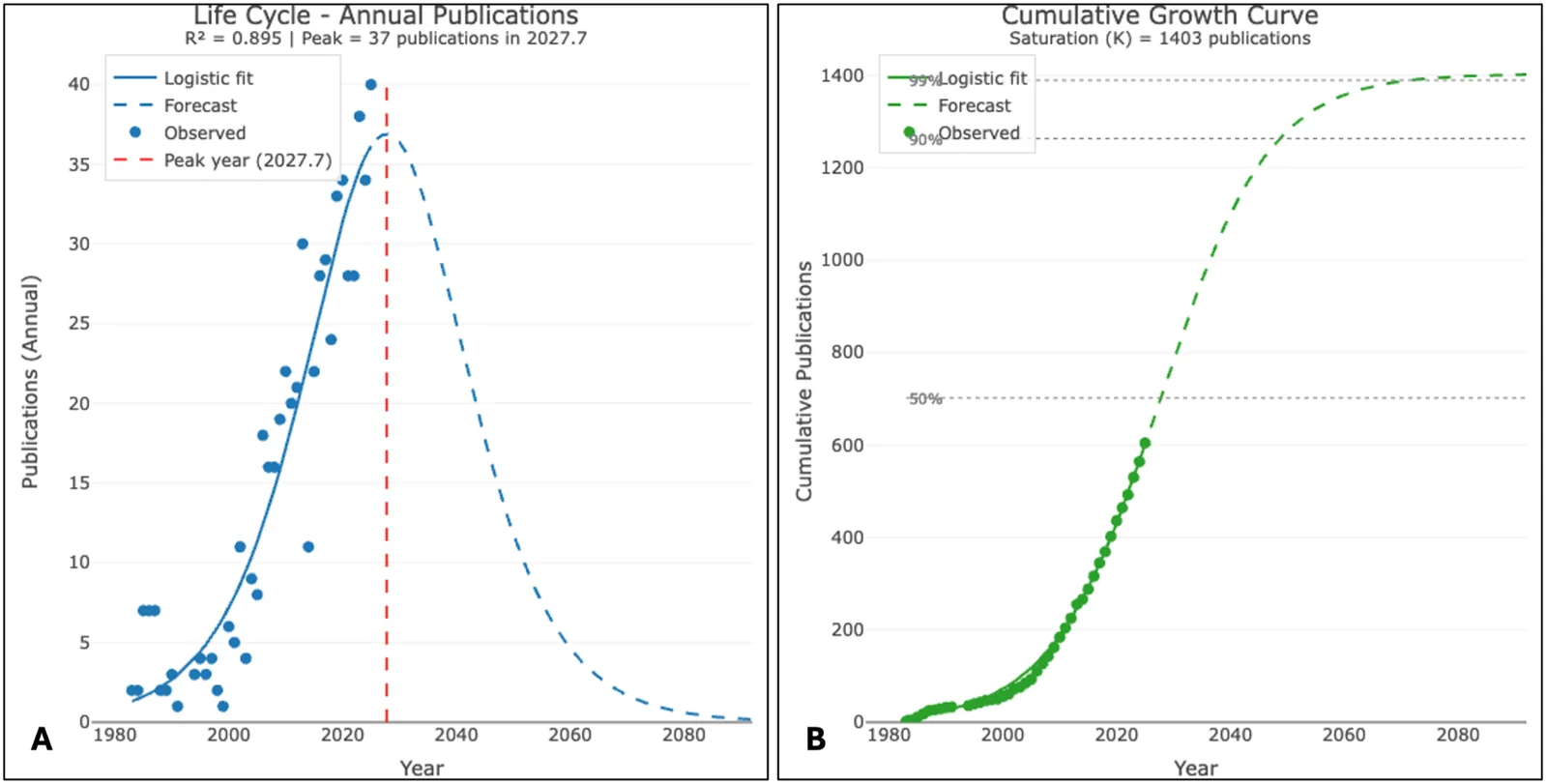
Life cycle of annual scientific production **(A)** and the cumulative growth curve **(B)** on bioactivity research in *G. frondosa*.

### 3.2 Most productive countries

The geographical distribution of scientific production on the bioactivity of *Grifola frondosa* demonstrates a pronounced concentration of English-language, Scopus-indexed publications in East Asia, with China and Japan emerging as the leading contributors within the analyzed dataset. China ranked first with 238 publications, followed by Japan with 116 publications. This regional concentration is consistent with the longstanding scientific and biomedical interest in *G. frondosa* as an edible and medicinal mushroom in East Asia, where investigations have extensively addressed its bioactive polysaccharides, immunomodulatory properties, antitumor activity, metabolic effects, and other functional bioactivities. The United States ranked third with 70 publications, followed by Taiwan and South Korea with 37 and 29 publications, respectively. Collectively, the marked productivity of these countries suggests that the global knowledge base has been shaped predominantly by research systems in regions where medicinal mushroom research has historically intersected with food science, natural-product pharmacology, biotechnology, and complementary medicine. However, these country-level rankings should be interpreted as indicators of bibliographic visibility within the English-language Scopus-indexed literature rather than as definitive measures of total national research activity, because studies published in local-language journals or in sources not indexed by Scopus may not be represented in the present dataset.

Scientific production outside the leading Asian countries was more dispersed and substantially lower in the analyzed corpus. Italy was the most productive European country, with 17 publications, followed by Spain and Germany with 14 publications each, while Argentina and Russia contributed 12 and 11 publications, respectively. Although this distribution indicates a marked asymmetry in indexed scientific output, lower publication counts should not be equated directly with lower underlying research activity. Differences in language of publication, national publishing practices, journal inclusion in international databases, and regional indexing coverage may influence the visibility of research from particular countries. Accordingly, **Figure 6** should be interpreted as depicting the geographical distribution of the retrieved and eligible Scopus-indexed literature rather than the complete global distribution of *G. frondosa* bioactivity research.

**Figure 6:**
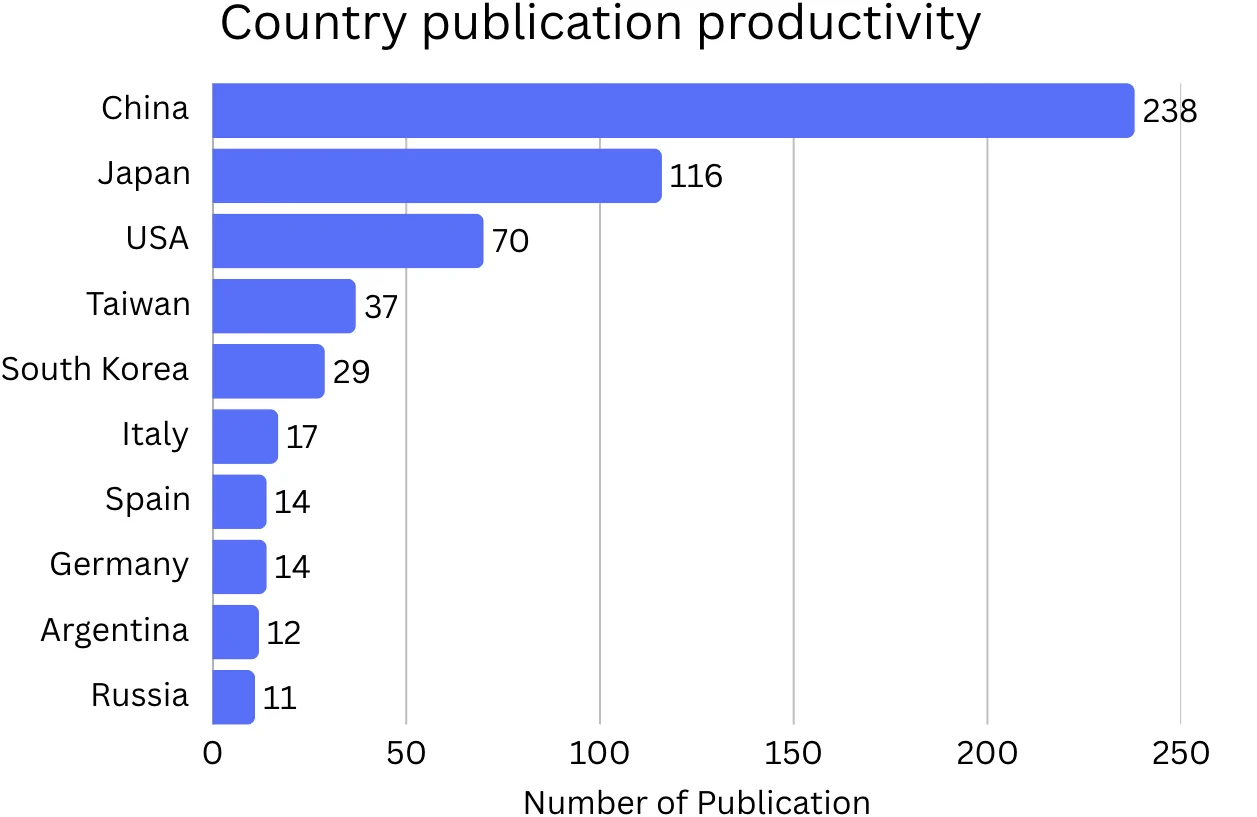
Country-specific scientific production of bioactivity research in *G. frondosa*.

### 3.3 Most relevant authors

**Table 1** identifies a strongly Japan-centered authorship structure in *G. frondosa* bioactivity research. Naohito Ohno ranks first with 39 publications, followed by Hiroaki Nanba with 32 and Toshiro Yadomae with 30. Most of the leading authors are affiliated with Japanese institutions, particularly Tokyo University of Pharmacy and Life Sciences and Kobe Pharmaceutical University, indicating that Japan has played a foundational role in advancing research on maitake bioactive compounds.

**Table 1:** Top 10 authors publishing on bioactivity research in *G. frondosa* (1980-2025).

Rank	Author name	Affiliation	Country	TP
1^st^	Ohno, Naohito	Tokyo University of Pharmacy and Life Sciences	Japan	39
2^nd^	Nanba, Hiroaki	Kobe Pharmaceutical University	Japan	32
3^rd^	Yadomae, Toshiro	Tokyo University of Pharmacy and Life Sciences	Japan	30
4^th^	Adachi, Yoshiyuki	Tokyo University of Pharmacy and Life Sciences	Japan	21
5^th^	Kodama, Noriko	Kobe Pharmaceutical University	Japan	21
6^th^	Oikawa, Shozo	Nippon Beet Sugar Mfg. Co., Ltd.	Japan	20
7^th^	Sato, Kichiro	Nippon Beet Sugar Mfg. Co., Ltd.	Japan	18
8^th^	Cui, Feng-Jie	Jiangsu University	China	16
9^th^	Suzuki, Iwao	Tokyo University of Pharmacy and Life Sciences	Japan	16
10^th^	Yang, Liuqing	Jiangsu University	China	16

TP, Total publication.

The dominance of Japanese authors suggests sustained expertise in the biochemical, pharmacological, and immunomodulatory investigation of *G. frondosa*, especially its polysaccharides and β-glucan fractions. The inclusion of Feng-Jie Cui and Liuqing Yang from Jiangsu University also reflects China’s growing contribution to the field. Overall, the authorship pattern shows that productivity is concentrated among a small group of specialized researchers and institutions, with East Asia serving as the principal center of knowledge production in *G. frondosa* bioactivity research.

### 3.4 Most relevant sources

The table shows that *G. frondosa* bioactivity research is concentrated in journals focused on medicinal mushrooms, biopolymers, pharmacology, food chemistry, and functional foods (**Table 2**). The International Journal of Medicinal Mushrooms ranks first by total publications, with 64 papers and 1,336 citations, indicating its central role as the main disciplinary outlet for this topic. The International Journal of Biological Macromolecules follows with 43 papers but has a higher citation impact, with 1,797 total citations, an average citation per paper of 41.79, and the highest h-index among the listed journals.

**Table 2:** Top 10 Scopus-indexed journals publishing bioactivity research in *G. frondosa*.

Rank	Journal title	CiteScore 2025	Publisher	TP	TC	ACP	h-index
1^st^	International Journal of Medicinal Mushrooms	3.3	Begell House	64	1,336	20.87	17
2^nd^	International Journal of Biological Macromolecules	11.4	Elsevier	43	1,797	41.79	26
3^rd^	Chemical and Pharmaceutical Bulletin	2.4	Pharmaceutical Society of Japan	21	1,236	58.85	15
4^th^	Carbohydrate Polymers	23.1	Elsevier	20	1,514	75.70	19
5^th^	Food Chemistry	17.5	Elsevier	18	1,524	84.66	14
6^th^	Mycoscience	2.5	The Mycological Society of Japan	11	182	16.54	6
7^th^	Journal of Medicinal Food	4.0	SAGE	10	476	47.60	10
8^th^	Food and Function	11.5	Royal Society of Chemistry	9	509	56.55	8
9^th^	Journal of Agricultural and Food Chemistry	9.5	American Chemical Society	9	540	60.00	8
10^th^	Biological and Pharmaceutical Bulletin	3.1	Pharmaceutical Society of Japan	8	720	90.00	7

TP, Total publication; TC, Total citation; ACP, Average citation per paper.

The citation indicators suggest that journals publishing fewer papers may still exert substantial influence. Biological and Pharmaceutical Bulletin records the highest average citation per paper at 90.00, followed by Food Chemistry at 84.66 and Carbohydrate Polymers at 75.70, reflecting the strong impact of studies on polysaccharides, β-glucans, bioactive macromolecules, antioxidant activity, and pharmacological properties. Overall, the source distribution indicates that the field is interdisciplinary, linking mycology, natural products chemistry, food science, pharmaceutical sciences, and biomacromolecular research.

### 3.5 Most relevant institutions

The institutional distribution of *Grifola frondosa* bioactivity research from 1980 to 2025 shows a marked concentration of scientific productivity in East Asia, particularly China and Japan (**Table 3**). The Ministry of Education of the People’s Republic of China ranked first with 47 publications, followed by Tokyo University of Pharmacy and Life Sciences with 41 and Kobe Pharmaceutical University with 37. Jiangsu University ranked fourth with 35 publications, while Tianjin University of Science & Technology contributed 29. This pattern indicates that the research landscape is supported by both national-level research structures and specialized universities with established expertise in pharmaceutical sciences, food science, biotechnology, and natural-product research. The strong representation of Tokyo University of Pharmacy and Life Sciences and Kobe Pharmaceutical University is consistent with Japan’s long-standing contribution to the pharmacological and immunological investigation of *G. frondosa*, whereas the prominence of several Chinese institutions reflects the increasing visibility of China in research on mushroom-derived bioactive compounds, polysaccharides, cultivation technologies, and functional food applications. Nippon Beet Sugar Manufacturing Co., Ltd., with 22 publications, further demonstrates the participation of industry-affiliated research organizations in the field, suggesting that investigations have extended beyond academic institutions toward applied and product-oriented research.

**Table 3:** Top 10 institutions publishing bioactivity research in *G. frondosa* (1980-2025).

Rank	Affiliation	Country	TP
1^st^	Ministry of Education of the People’s Republic of China	China	47
2^nd^	Tokyo University of Pharmacy and Life Sciences	Japan	41
3^rd^	Kobe Pharmaceutical University	Japan	37
4^th^	Jiangsu University	China	35
5^th^	Tianjin University of Science & Technology	China	29
6^th^	Nippon Beet Sugar Manufacturing Co., Ltd.	Japan	22
7^th^	Fujian Agriculture and Forestry University	China	20
8^th^	National Taiwan University	Taiwan	14
9^th^	Shanghai Academy of Agricultural Sciences	China	14
10^th^	Jiangxi Provincial Engineering and Technology Center for Food Additives Bio-production	China	10

TP, Total publication.

The lower half of the ranking further illustrates the diversity of institutional participation within the East Asian research ecosystem. Fujian Agriculture and Forestry University contributed 20 publications, highlighting the role of agricultural and forestry institutions in linking fungal biology, cultivation, and bioactive-compound research. National Taiwan University ranked eighth with 14 publications, while the Shanghai Academy of Agricultural Sciences also recorded 14 publications, indicating continued contributions from both comprehensive universities and specialized agricultural research organizations. The Jiangxi Provincial Engineering and Technology Center for Food Additives Bio-production ranked tenth with 10 publications, reflecting the involvement of applied research centers focused on food processing, bioproduction, and value-added product development. Overall, six of the top ten institutions are based in China, three are located in Japan, and one is in Taiwan, demonstrating that institutional productivity is geographically concentrated within East Asia and spans pharmaceutical universities, agricultural institutions, government-affiliated research bodies, industrial organizations, and engineering-oriented research centers.

### 3.6 Scientific co-authorship and collaboration networks

The country-level co-authorship network (**Figure 7A**) reveals a highly centralized and internationally connected research structure in *G. frondosa* bioactivity studies. China occupies the largest node and has the highest number of collaborative links, indicating its dual role as the most productive and most internationally connected country in the field. Numerous connections extend from China to the United States, Japan, South Korea, Taiwan, Germany, Finland, Thailand, and Hong Kong, demonstrating its prominent position as a global collaboration hub. Japan constitutes the second major node and maintains strong collaborative relationships with the United States, Australia, France, and several European countries, reflecting its long-standing contribution to medicinal mushroom research. The United States serves as an intermediary node linking Asian and European research groups, suggesting an important bridging role in facilitating knowledge exchange and interdisciplinary cooperation. Smaller nodes representing countries such as Italy, Spain, Portugal, Argentina, Brazil, Turkey, and Honduras indicate broader geographic participation, although these countries contribute with lower publication outputs and fewer collaborative links.

**Figure 7:**
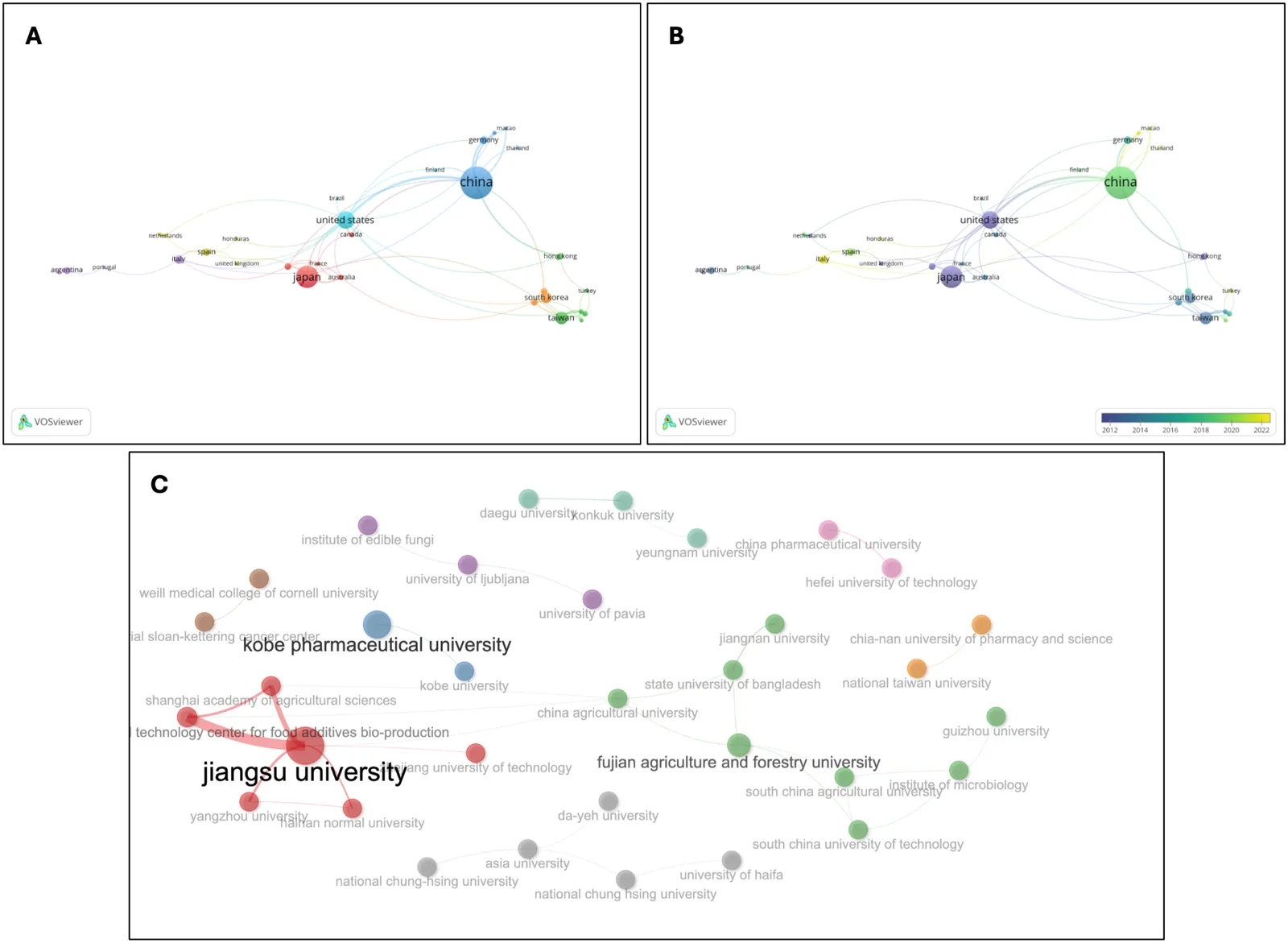
Co-authorship on bioactivity research in *G. frondosa* among countries and collaboration through **(A)** network analysis, **(B)** overlay visualization, and **(C)** institutional collaboration. In the network visualizations, each node represents a country or institution, with node size proportional to its relative contribution/weight in the network. Lines connecting nodes represent co-authorship or collaborative relationships, with stronger links indicating greater collaboration between connected entities. In **(A, C)**, node colors distinguish clusters of closely connected countries or institutions identified by the network clustering algorithm; the colors are categorical and do not indicate relative performance or ranking. In the overlay visualization **(B)**, node color represents the average publication year, progressing from darker blue/purple tones for earlier contributions through green to yellow for more recent contributions. Spatial proximity between nodes generally reflects stronger relatedness within the network.

The overlay visualization (**Figure 7B**) offers temporal insights into the evolution of international collaborations. Earlier collaborative activities, depicted by darker colors, are primarily concentrated in Japan, the United States, and several European countries, indicating that these nations played pioneering roles during the initial phases of *G. frondosa* bioactivity research. In contrast, countries shown in lighter green and yellow, particularly China, Germany, Thailand, and several East Asian partners, represent more recent and rapidly expanding collaborations. This temporal progression illustrates a gradual shift in scientific leadership from Japanese- and American-centered research networks toward a collaborative landscape increasingly dominated by China. The rising prominence of China aligns with substantial investments in medicinal mushroom research, functional food development, natural product chemistry, and biotechnology. Additionally, the formation of new collaborative links involving South Korea, Taiwan, and Southeast Asian countries indicates ongoing regional expansion of research activities focused on the pharmacological, immunomodulatory, antioxidant, and polysaccharide-related properties of *G. frondosa*.

The institutional collaboration network (**Figure 7C**) demonstrates that *G. frondosa* bioactivity research is structured into multiple research clusters, each representing distinct institutional partnerships and thematic specializations. Several highly connected institutional nodes serve as central hubs, indicating the presence of leading organizations that coordinate collaborative activities within their respective clusters. The red and green clusters comprise the largest and most interconnected institutions, reflecting strong internal collaboration and sustained research productivity. In contrast, smaller clusters with limited connections represent specialized research groups operating within narrower scientific domains. The relatively sparse interconnections between clusters indicate that institutional collaborations remain partially fragmented despite the expansion of international co-authorship networks. These collaborative structures likely reflect differences in research priorities, such as medicinal chemistry, mushroom polysaccharides, β-glucan characterization, immunomodulation, food science, cancer therapeutics, and functional food applications. The coexistence of several independent yet productive institutional groups underscores both the multidisciplinary nature of *G. frondosa* bioactivity research and the potential for future integration of expertise across geographical and disciplinary boundaries.

### 3.7 Keyword co-occurrence network analysis

The keyword co-occurrence network (**Figure 8A**) indicates that *G. frondosa* functions as the central organizing concept of the field, with strong links to “medicinal mushroom,” “β-glucan,” “antioxidant activity,” and “antitumor activity.” The network is structured around several thematic clusters: a medicinal and immunological cluster associated with “immunomodulation,” “inflammation,” “cytokines,” and “apoptosis”; a β-glucan-centered cluster linked to “macrophage,” “dectin-1,” “TNF-α,” and “antitumor glucan”; and a biochemical-functional cluster connected to “structural characterization,” “purification,” “cytotoxicity,” “hypoglycemic,” and “gut microbiota.” The observed patterns indicate that research in this field is primarily focused on polysaccharides, immune modulation, anticancer mechanisms, antioxidant activity, and functional food applications. The prominence of these topics within the network reflects their frequency and connectivity in the literature; however, this should not be interpreted as comparative evidence of biological significance or therapeutic efficacy.

**Figure 8:**
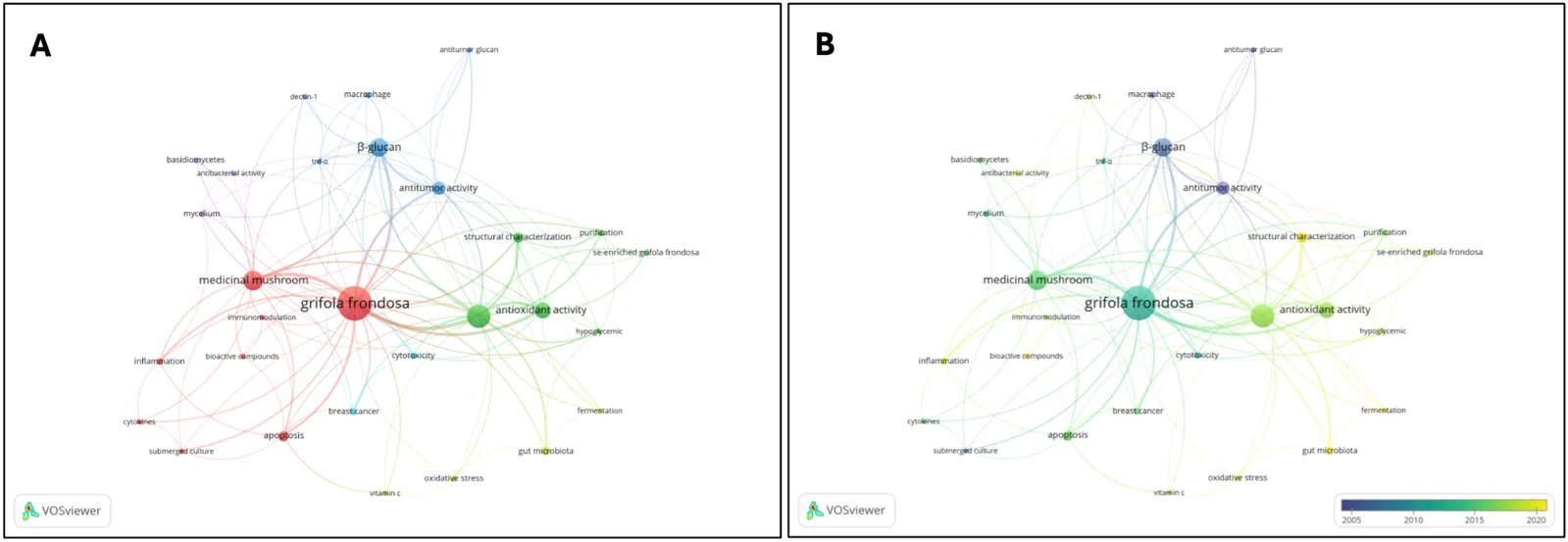
Keyword co-occurrence through **(A)** network visualization and **(B)** overlay visualization of bioactivity research in *G. frondosa.* Each node represents a keyword, and node size is proportional to its frequency of occurrence in the analyzed literature. Links between nodes indicate keyword co-occurrence, with stronger links representing greater co-occurrence strength. In the network visualization **(A)**, colors represent clusters of keywords that frequently co-occur and therefore indicate major thematic groupings; cluster colors are categorical and do not represent ranking or importance. In the overlay visualization **(B)**, node color represents the average publication year associated with each keyword, with darker blue/purple tones indicating earlier research topics, green representing intermediate periods, and yellow indicating more recent research topics. The spatial proximity of keywords reflects their relative strength of association within the co-occurrence network.

The overlay visualization shows a temporal progression in research emphasis. Earlier studies were more closely associated with core themes such as “β-glucan,” “antitumor activity,” and immunological mechanisms, reflecting foundational work on maitake-derived glucans and host immune responses. More recent keywords, shown in lighter green to yellow tones, include “structural characterization,” “antioxidant activity,” “purification,” “fermentation,” “gut microbiota,” “oxidative stress,” and “vitamin C,” indicating a shift toward mechanistic characterization, processing optimization, and broader nutraceutical or functional food applications (**Figure 8B**). This evolution suggests that *G. frondosa* bioactivity research has expanded from classical antitumor and immunomodulatory investigations toward more integrative studies involving molecular characterization, bioactive compound purification, metabolic effects, and interactions with the gut microbiome.

### 3.8 Trend and burning topics

The temporal evolution of research topics reveals a distinct progression in the scientific focus of *Grifola frondosa* bioactivity research over the past four decades (**Figure 9**). In the earliest phase, from the mid-1980s to the early 2000s, research primarily addressed fundamental bioactive components and their biological functions, with particular emphasis on “β-1,3-glucan,” “β-glucan,” “antitumor activity,” and “macrophage.” These topics persisted over the longest periods, underscoring their foundational role in establishing the medicinal relevance of *G. frondosa*. Early studies concentrated on isolating and characterizing polysaccharides and glucan fractions, as well as evaluating their capacity to stimulate macrophages, modulate cytokine production, and induce antitumor responses. The frequent appearance of terms such as “TNF-α,” “submerged culture,” and “basidiomycetes” indicates an initial focus on immunological mechanisms, cultivation techniques, and fungal biology. The subsequent emergence of “cytotoxicity,” “Grifola frondosa,” and “medicinal mushroom” in the late 2000s and early 2010s marks a transition toward broader pharmacological assessments and the recognition of maitake as a clinically significant medicinal mushroom.

**Figure 9:**
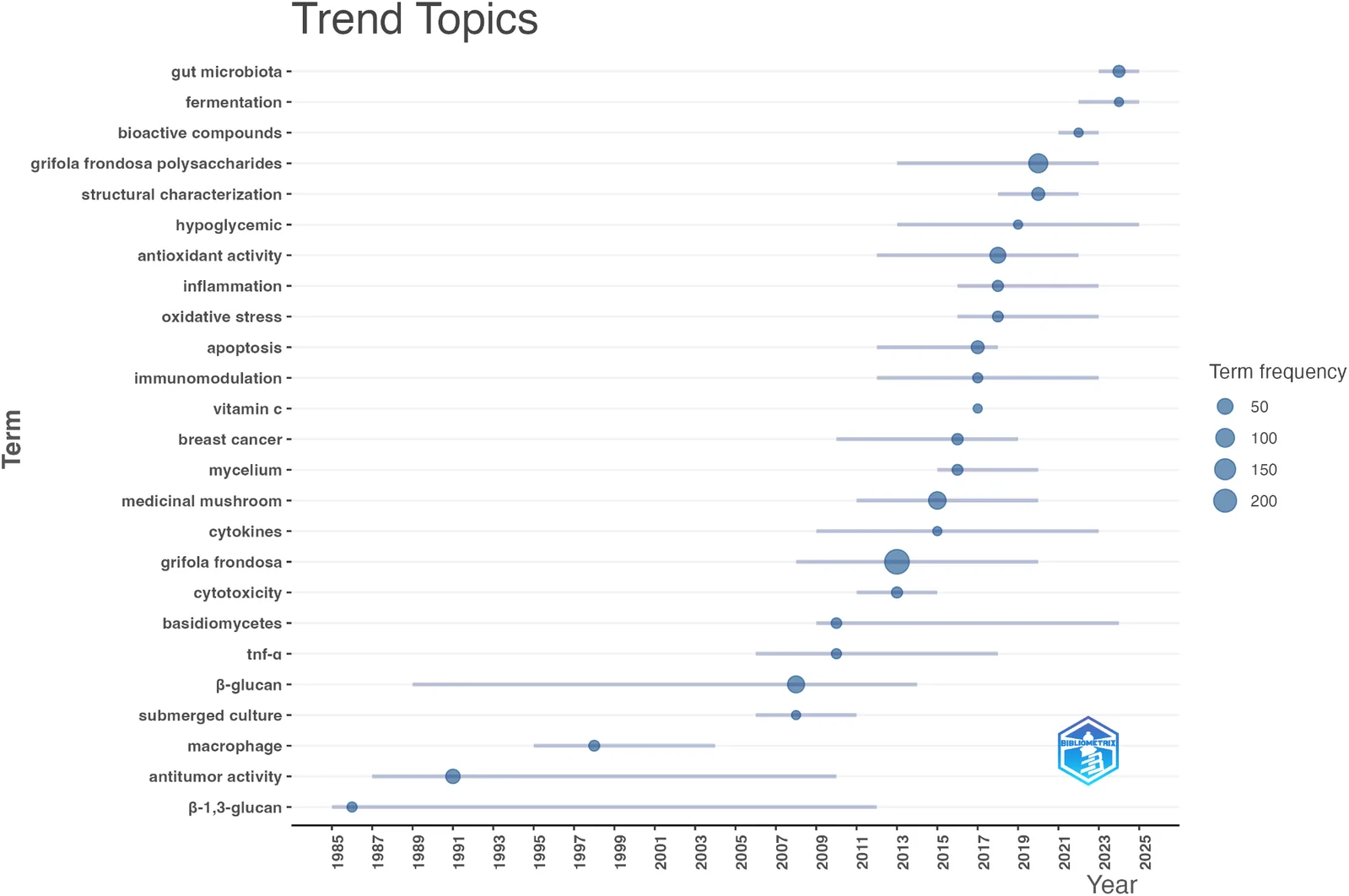
Topics studied over time in relation to bioactivity research in *G. frondosa*.

Since approximately 2013, the field has experienced significant thematic diversification, as evidenced by the rising prominence of topics such as “immunomodulation,” “apoptosis,” “oxidative stress,” “inflammation,” and “antioxidant activity.” The large bubble sizes for “Grifola frondosa,” “Grifola frondosa polysaccharides,” and “antioxidant activity” reflect their high frequency and central significance during this period. After 2018, new topics such as “structural characterization,” “bioactive compounds,” “fermentation,” and “gut microbiota” have emerged, indicating a shift toward advanced analytical methodologies and applications in functional foods. The introduction of research on “hypoglycemic” effects and gut microbiota highlights growing interest in metabolic health, nutraceutical development, and host–microbiome interactions. Additionally, the increased focus on structural characterization and purification demonstrates that recent studies aim to elucidate structure–activity relationships, identify specific bioactive molecules, and clarify the molecular mechanisms underlying the diverse pharmacological activities of *G. frondosa*.

The word cloud shows that *G. frondosa* is the dominant term, confirming that the literature is strongly centered on this medicinal mushroom as the principal organism of interest (**Figure 10**). The high prominence of “*G. frondosa* polysaccharides,” “β-glucan,” and “medicinal mushroom” indicates that bioactivity research in this field is largely driven by investigations of polysaccharide-rich fractions, particularly β-glucans, as key bioactive components associated with pharmacological and nutraceutical properties.

**Figure 10:**
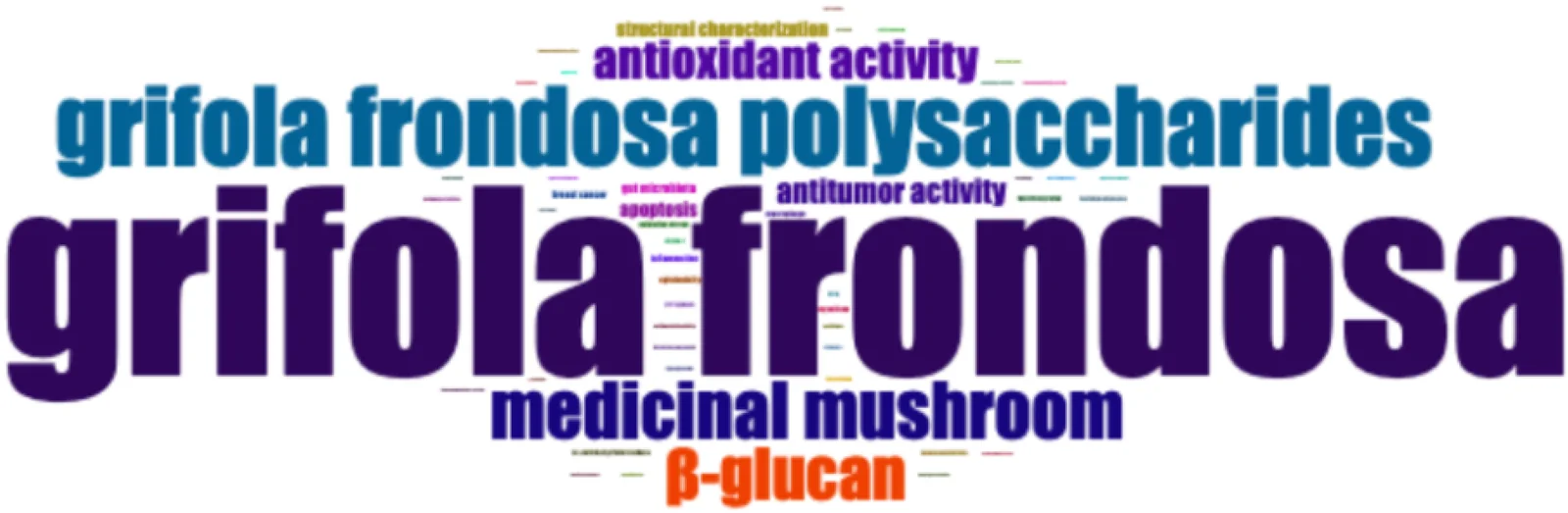
Word cloud visualizes the most frequently occurring terms in bioactivity research in *G. frondosa*, with themes in the field.

The visible presence of terms such as “antioxidant activity,” “antitumor activity,” and “structural characterization” reflects the major thematic directions of the field. These terms suggest that studies have focused not only on identifying biological effects but also on linking these activities to chemical composition, molecular structure, and functional mechanisms. Thus, the word cloud highlights an interdisciplinary research landscape integrating medicinal mycology, polysaccharide chemistry, antioxidant and anticancer bioactivity, and functional food development.

### 3.9 Conceptual structure mapping

The strategic thematic map shows that bioactivity research on *G. frondosa* is anchored by several basic themes with high centrality but comparatively lower density, particularly “*Grifola frondosa*,” “*G. frondosa* polysaccharides,” “structural characterization,” “gut microbiota,” “antioxidant activity,” and “inflammation”, as shown in **Figure 11A**. These themes represent the core intellectual foundation of the field, indicating that current research is strongly organized around polysaccharide bioactivity, antioxidant and anti-inflammatory mechanisms, and emerging links with gut microbiota modulation. Their position in the basic themes quadrant suggests that these topics are highly relevant to the broader research domain but remain open to further conceptual and methodological development.

**Figure 11:**
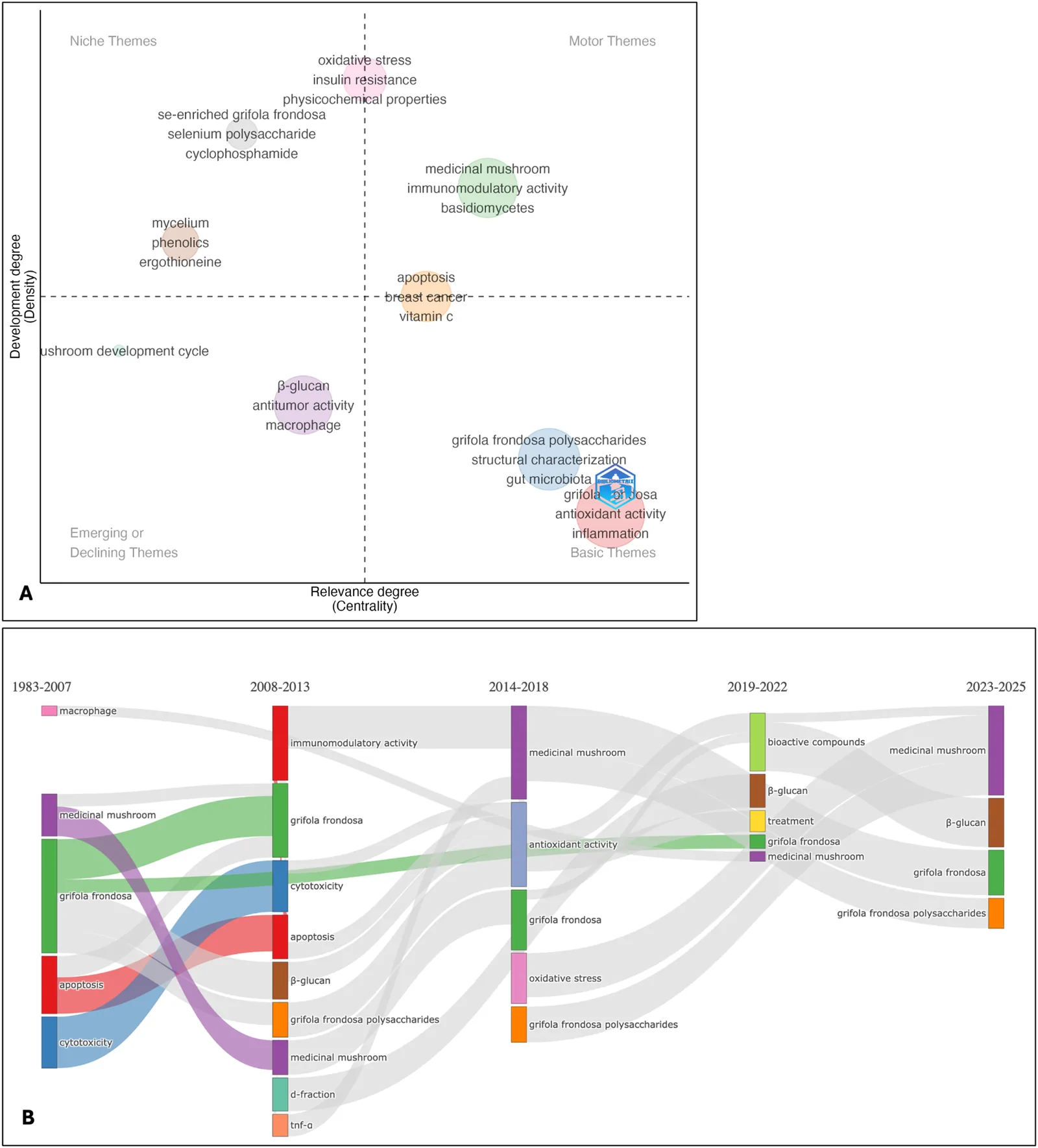
Strategic thematic map **(A)** and thematic evolution **(B)** of bioactivity research in *G. frondosa*.

The motor themes quadrant contains “medicinal mushroom,” “immunomodulatory activity,” and “basidiomycetes,” indicating a mature and well-developed thematic area with strong relevance to the overall field (**Figure 11A**). This reflects the central role of *G. frondosa* within medicinal mushroom research and its established association with immune-related biological effects. In contrast, the niche themes quadrant includes specialized topics such as “oxidative stress,” “insulin resistance,” “physicochemical properties,” “Se-enriched *G. frondosa*,” “selenium polysaccharide,” “cyclophosphamide,” “mycelium,” “phenolics,” and “ergothioneine.” These themes are internally developed but less central, suggesting focused subfields related to metabolic regulation, selenium enrichment, fungal metabolites, and adjunctive therapeutic applications.

The thematic evolution diagram shows a transition from early research centered on “medicinal mushroom,” “*Grifola frondosa*,” “apoptosis,” “cytotoxicity,” and “macrophage” during 1983–2007 toward more mechanistic and compound-specific themes in later periods (**Figure 11B**). From 2008-2018, themes such as “β-glucan,” “D-fraction,” “TNF-α,” “immunomodulatory activity,” “antioxidant activity,” “oxidative stress,” and “*G. frondosa* polysaccharides” became prominent, reflecting increasing emphasis on defined bioactive fractions and immune-related pathways. In 2019–2025, the field further shifted toward “bioactive compounds,” “β-glucan,” “treatment,” “medicinal mushroom,” “*G. frondosa*,” and “*G. frondosa* polysaccharides,” indicating growing attention to purified constituents, translational relevance, and functional applications of maitake-derived bioactive molecules.

The factorial analysis through multidimensional scaling (MDS) reveals four major conceptual clusters that collectively define the intellectual structure of bioactivity research on *G. frondosa* (**Figure 12**).

**Figure 12:**
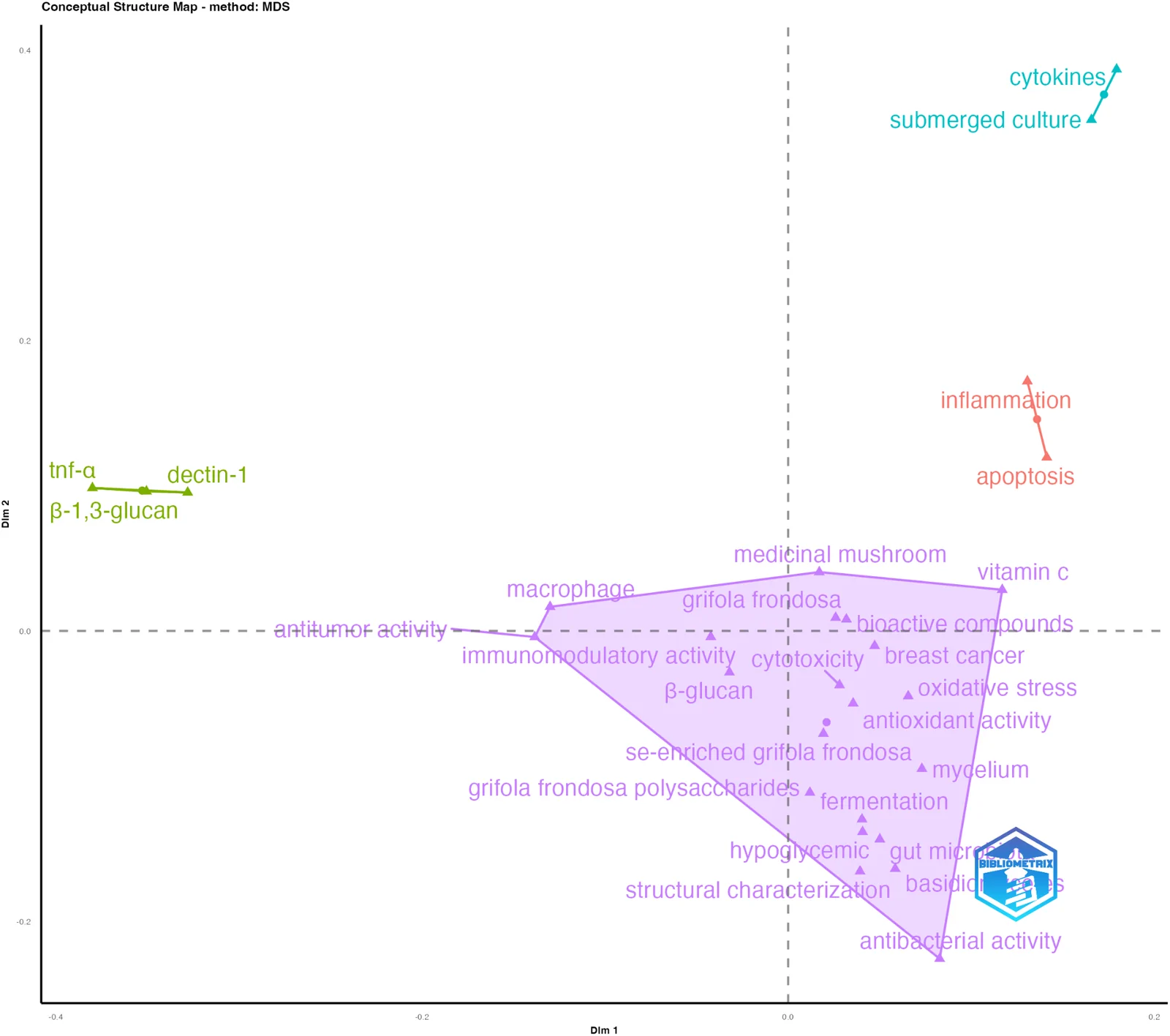
Factorial analysis through four-clustered multidimensional scaling (MDS) of bioactivity research in *G. frondosa*.

The largest cluster, primarily situated in the central and lower-right regions of the conceptual space, includes terms such as “*Grifola frondosa*,” “β-glucan,” “*Grifola frondosa* polysaccharides,” “structural characterization,” “antioxidant activity,” “cytotoxicity,” “oxidative stress,” “gut microbiota,” “hypoglycemic,” “fermentation,” “bioactive compounds,” and “basidiomycetes.” The close spatial proximity of these terms suggests strong conceptual associations and frequent co-occurrence in the literature. This cluster delineates the principal research domain, with a focus on the isolation, characterization, and functional assessment of bioactive compounds, particularly polysaccharides and β-glucans. The presence of gut microbiota, hypoglycemic activity, and antioxidant activity within this cluster illustrates the field’s progression from traditional medicinal applications to research on metabolic health, nutraceutical development, and microbiome-mediated mechanisms.

A second cluster, located in the upper-left quadrant, comprises “β-1,3-glucan,” “dectin-1,” and “TNF-α,” collectively representing an immunological and receptor-mediated signaling theme. The relatively isolated position of this cluster suggests a specialized research domain focused on the molecular mechanisms of immune activation. β-1,3-glucans are identified as the principal bioactive polysaccharides of *G. frondosa*, and their interactions with pattern-recognition receptors such as dectin-1 promote cytokine production and macrophage activation. The close association of TNF-α with these terms underscores the significance of inflammatory signaling pathways and innate immune responses in the biological activity of maitake-derived glucans. The terms “antitumor activity,” “macrophage,” and “immunomodulatory activity,” positioned between this immune cluster and the central core, indicate that these themes serve as conceptual bridges linking classical immunological studies with broader pharmacological and therapeutic investigations.

The upper-right region of the MDS map contains a third cluster, consisting of “cytokines” and “submerged culture,” which reflects a research direction integrating cultivation technology with immunological outcomes. The association between submerged culture systems and cytokine-related studies indicates that production methods and cultivation conditions are increasingly examined for their effects on bioactive compound synthesis and biological activity. A fourth cluster, represented by “apoptosis” and “inflammation,” occupies an intermediate position and signifies a biomedical and mechanistic research domain focused on cellular responses and disease-related pathways. The spatial separation of these terms from the central polysaccharide cluster suggests that apoptosis and inflammatory regulation are specialized therapeutic applications rather than general research themes. Collectively, the four-cluster MDS structure demonstrates that *G. frondosa* bioactivity research has progressed from early immunological and antitumor studies to a multidisciplinary field encompassing polysaccharide chemistry, molecular immunology, functional foods, microbial interactions, metabolic health, and therapeutic mechanisms. The central placement of *G. frondosa*, β-glucan, and polysaccharide-related terms indicates that these topics remain the conceptual foundation for emerging research areas such as gut microbiota modulation, antioxidant activity, and bioactive compound characterization.

## 4 Discussion

### 4.1 Evolution and maturation of *G. frondosa* bioactivity research

#### 4.1.1 From traditional medicinal mushroom research to modern bioactivity investigations

The progression of *G. frondosa* bioactivity research illustrates a shift from traditional medicinal applications to a multidisciplinary scientific domain encompassing immunology, pharmacology, natural products chemistry, and functional food science. Maitake has historically been consumed in China and Japan as both a medicinal and edible mushroom, with traditional beliefs attributing to its properties such as promoting longevity, enhancing vitality, and increasing resistance to disease. Scientific studies in the 1980s and 1990s primarily aimed to substantiate these ethnomedicinal claims by investigating the medicinal properties of crude extracts and polysaccharide-rich fractions. Current scientometric analyses indicate that early research focused on themes such as “β-glucan,” “macrophage,” “antitumor activity,” and “immunomodulation,” highlighting an initial emphasis on immune regulation and cancer-related applications. Foundational studies identified D-fraction and grifolan as principal bioactive constituents that stimulate macrophages, natural killer cells, T lymphocytes, and cytokine production, thereby establishing the immunological relevance of maitake polysaccharides (Kodama et al., 2003; Švagelj et al., 2012). β-Glucans derived from *G. frondosa* have since been recognized as significant biological response modifiers that activate innate immune receptors, including dectin-1 and complement receptor 3, leading to enhanced antitumor and immunostimulatory effects (Herre et al., 2004; Goodridge et al., 2009; Chan et al., 2009). Since the early 2000s, there has been a marked increase in publications, reflecting growing scientific interest in medicinal mushrooms as sources of therapeutic agents and nutraceuticals. Recent research has broadened to encompass antioxidant, anti-inflammatory, hypoglycemic, neuroprotective, and metabolic effects, as well as modulation of the gut microbiota and applications in functional foods (He et al., 2018; Wu et al., 2021; Murphy et al., 2020). Additionally, advancements in analytical chemistry and molecular biology have enabled detailed structural characterization of bioactive polysaccharides and clarification of their mechanisms of action, supporting the transformation of *G. frondosa* from a traditional medicinal mushroom to a scientifically validated source of therapeutic and nutraceutical compounds (Wasser, 2014; Zhong et al., 2023; Camilleri et al., 2024).

#### 4.1.2 Scientific growth, citation impact, and research maturity

The publication and citation patterns identified in this study demonstrate that *G. frondosa* bioactivity research has evolved from an emerging scientific niche into a mature and increasingly influential research domain. The limited publication output prior to the early 2000s likely reflects the small number of research groups focused on medicinal mushrooms during that period. In contrast, the substantial increase in annual publications after 2005 aligns with growing global interest in natural products, complementary medicine, functional foods, and mushroom-derived therapeutics. Citation peaks observed in the early 2000s and again between 2017 and 2021 indicate that several foundational studies significantly shaped subsequent research directions, particularly those related to β-glucan-mediated immunomodulation, antitumor activity, and polysaccharide characterization. Highly cited investigations on maitake D-fraction, immune activation, and biological response modifiers established the mechanistic basis for many contemporary studies (Vetvicka and Vetvickova, 2014; Vetvicka et al., 2021). The logistic growth model developed in this study further suggests that the field is entering a mature developmental phase, characterized by sustained publication output and increasing thematic diversification. Comparable life-cycle trajectories have been documented in other medicinal mushroom fields, such as *Ganoderma lucidum* and *Lentinula edodes*, which have progressed from traditional medicinal uses to multidisciplinary research encompassing immunology, oncology, food science, and biotechnology (Patel and Goyal, 2012; Wasser, 2014). The rising citation impact over the past decade likely reflects broader recognition of mushroom-derived β-glucans as clinically relevant immunomodulators and functional ingredients. Additionally, the emergence of recent topics including gut microbiota, oxidative stress, structural characterization, and fermentation indicates that the field continues to generate new scientific directions despite its maturity. The ongoing expansion of these themes demonstrates that *G. frondosa* research remains dynamic and continues to attract interdisciplinary interest from pharmacology, nutrition, biotechnology, and medical sciences (Ren et al., 2012; Valverde et al., 2015; Murphy et al., 2020). Therefore, the observed publication trajectory, citation performance, and thematic evolution collectively indicate that *G. frondosa* bioactivity research has progressed from an exploratory stage to a mature field with established conceptual foundations and emerging translational opportunities.

#### 4.1.3 Geographical concentration of research productivity

The strong geographical concentration of English-language, Scopus-indexed *G. frondosa* bioactivity research in East Asia appears to reflect the combined influence of historical traditions, cultural familiarity with medicinal mushrooms, established research infrastructure, and sustained investment in biotechnology and functional food research. Within the analyzed corpus, China and Japan lead publication output, institutional productivity, influential authorship, and international collaboration, underscoring their central roles in the indexed development of the field. This prominence is consistent with the long-standing use of medicinal mushrooms in traditional Chinese medicine and Japanese Kampo medicine, where maitake has been valued as both a dietary and therapeutic resource. Japanese researchers were particularly influential in the early immunological and pharmacological investigation of maitake polysaccharides, including D-fraction and β-glucan-mediated immune responses, whereas China has more recently expanded its contribution through research on medicinal mushroom cultivation, natural-product chemistry, polysaccharide characterization, biotechnology, and functional food development. The growth of commercial mushroom industries and nutraceutical markets in East Asia has further supported investigations aimed at improving cultivation technologies, optimizing polysaccharide production, and developing value-added therapeutic and functional products (Roupas et al., 2012; Valverde et al., 2015). The presence of specialized institutions and dedicated research programs has likewise facilitated sustained productivity and international collaboration. However, the apparent geographical concentration should be interpreted cautiously because the present analysis was restricted to English-language publications indexed in Scopus. Consequently, comparatively low representation from Africa, Latin America, Southeast Asia, and other regions may reflect not only differences in funding, infrastructure, and access to advanced analytical technologies, but also variation in local-language publishing practices, regional journal coverage, and database indexing. Similar East Asian dominance has been reported in medicinal mushroom research more broadly, where cultural traditions, industrial capacity, and established programs in fungal biotechnology have contributed to high scientific visibility (Lull et al., 2005; Wasser, 2014). Thus, the lower publication counts observed for many low- and middle-income countries should not be interpreted solely as evidence of limited research activity or interest, because relevant work published in local or non-Scopus-indexed sources may be underrepresented. The geographical findings are therefore best understood as patterns of bibliographic visibility within the English-language Scopus-indexed literature rather than as a complete representation of global *G. frondosa* research activity, highlighting the value of future multilingual and multi-database analyses alongside stronger international collaboration and technology transfer to achieve a more inclusive assessment of the field.

### 4.2 Knowledge production and collaborative research networks

#### 4.2.1 Scientific leadership and authorial concentration

The development of *G. frondosa* bioactivity research has been strongly influenced by a relatively small group of highly productive researchers, particularly those based in Japan, who established many of the concepts and experimental approaches that continue to shape the field today. The bibliometric results demonstrate that authors such as Naohito Ohno, Hiroaki Nanba, Toshiro Yadomae, Yoshiyuki Adachi, and Noriko Kodama have played central roles in advancing knowledge on maitake polysaccharides, particularly β-glucan fractions, and their biological activities. Early studies conducted by these researchers provided some of the first evidence that *G. frondosa* polysaccharides could stimulate macrophages, enhance cytokine production, and activate natural killer cells, thereby establishing the immunological basis of maitake bioactivity (Adachi et al., 1994; Kodama et al., 2003). These pioneering investigations also helped define the major research themes that continue to dominate the field, including immunomodulation, antitumor activity, and polysaccharide-mediated immune responses. The concentration of productive authors within a limited number of institutions suggests the existence of specialized research groups that have maintained long-term expertise and sustained research productivity over several decades. Such continuity has enabled the gradual accumulation of knowledge, allowing successive studies to build upon earlier findings and further refine the understanding of maitake bioactive compounds. More recently, the increasing contribution of Chinese researchers has expanded the field toward areas such as structural characterization, fermentation technology, and functional food applications, reflecting a shift toward broader multidisciplinary research. Consequently, the intellectual development of *G. frondosa* research has been largely driven by a combination of scientific leadership, institutional continuity, and sustained specialization, which together have shaped the conceptual foundations and future directions of the field (He et al., 2018; Vetvicka and Vetvickova, 2014; Wu et al., 2021).

#### 4.2.2 Institutional and international collaboration patterns

The collaboration networks identified in this study highlight the role of international partnerships and institutional interactions in the development of *G. frondosa* bioactivity research. China occupies a prominent position within the international collaboration network, consistent with its high publication output and substantial representation among productive institutions in the analyzed corpus. This pattern appears consistent with the broader expansion of Chinese research in medicinal mushrooms, natural-product chemistry, biotechnology, polysaccharide research, and functional food development reported in the literature (He et al., 2018; Wu et al., 2021). Japan and the United States are also prominent within the network, with their contributions spanning earlier and continuing investigations of *G. frondosa* bioactivity. Japanese institutions were particularly involved in early studies of D-fraction, β-glucan-mediated immunological responses, and antitumor activity, whereas researchers in the United States contributed to the clinical evaluation and translational investigation of maitake-derived compounds (Kodama et al., 2003; Deng et al., 2009). Although previous literature provides broader contextual evidence regarding national developments in medicinal mushroom research, biotechnology, and functional food science, the present collaboration analysis does not directly evaluate the socioeconomic, policy, or investment-related factors underlying these country-level patterns. Accordingly, the prominence of China, Japan, and the United States should be interpreted primarily in terms of their bibliographic representation and network connectivity within the analyzed Scopus-indexed corpus rather than as direct evidence of differences in national research capacity or investment. At the institutional level, the observed clusters suggest a degree of thematic specialization, with some research groups concentrating on immunomodulation and cancer biology and others emphasizing polysaccharide chemistry, fermentation technology, or functional food applications. The comparatively limited interconnections among several institutional clusters further suggest that the collaboration landscape remains partially fragmented. Such fragmentation may constrain opportunities for integrating basic, applied, and clinical research, although its direct effect on translational outcomes cannot be determined from co-authorship data alone. Strengthening multidisciplinary collaboration across immunology, pharmacology, biotechnology, nutrition, food science, and clinical research may therefore facilitate greater exchange of methods and expertise and support future translational investigations. This may be particularly relevant as contemporary studies increasingly examine gut microbiota modulation, metabolic regulation, and the biological and clinical potential of β-glucans, research areas that require expertise extending beyond conventional medicinal mushroom research (Murphy et al., 2020, 2022; Zhong et al., 2023).

### 4.3 Dominant scientific themes and mechanistic foundations

#### 4.3.1 Central role of β-glucans and polysaccharides

The present scientometric analysis reveals that polysaccharide fractions and β-glucans constitute the primary intellectual cornerstone of *G. frondosa* bioactivity research, as demonstrated by their prominent centrality within keyword co-occurrence networks, thematic evolution, strategic mapping, and factorial analyses. Their sustained prominence over several decades indicates that early investigations into their structural and functional properties have laid the foundation for subsequent research on immunomodulation, antitumor activity, antioxidant potential, and, more recently, gut microbiota interactions. This enduring scientific importance is fundamentally linked to their intricate molecular architecture and diverse pharmacological activities. Major bioactive polysaccharides of *G. frondosa*, particularly β-(1→3)-D-glucans with β-(1→6)-linked branches such as the D-fraction and grifolan, possess highly ordered triple-helical conformations that are essential for biological activity (Patel et al., 2021). Their molecular weight, branching patterns, solubility, and tertiary structure critically influence receptor binding affinity and immune recognition (Chen et al., 2022). Unlike conventional cytotoxic chemotherapeutic agents, β-glucans function primarily as biological response modifiers that enhance host immune responses rather than directly inducing tumor cell death (Murphy et al., 2020). This mechanism explains the strong conceptual associations observed in the present bibliometric analysis among β-glucans, macrophage activation, cytokine regulation, immunomodulation, and antitumor activity. As pathogen-associated molecular patterns (PAMPs), β-glucans are recognized by Dectin-1, a pattern-recognition receptor highly expressed on macrophages, dendritic cells, neutrophils, and monocytes (Herre et al., 2004).

Dectin-1 engagement activates spleen tyrosine kinase (Syk)-dependent signaling and the CARD9–NF-κB pathway, resulting in the production of pro-inflammatory cytokines including TNF-α, IL-1β, IL-6, and IL-12, which collectively promote antigen presentation, macrophage polarization, natural killer cell cytotoxicity, and T-cell activation (Mata-Martínez et al., 2022). Furthermore, cooperative signaling through complement receptor 3 (CR3), Toll-like receptors (TLRs), and other C-type lectin receptors amplifies both innate and adaptive immune responses, highlighting receptor-mediated signaling as a central mechanistic theme in β-glucan immunobiology (Reis et al., 2019). These immune pathways provide the biological basis for the extensive antitumor literature identified in the keyword network and thematic evolution analyses. Rather than exerting direct cytotoxicity, *G. frondosa* β-glucans suppress tumor progression by activating dendritic cells, macrophages, natural killer cells, and cytotoxic T lymphocytes while reshaping the cytokine milieu within the tumor microenvironment (Rossi et al., 2018). The emergence of contemporary themes, including structural characterization, structure–activity relationships, gut microbiota, and precision nutraceuticals, further demonstrates that current research has expanded upon rather than replaced the classical β-glucan paradigm. Consequently, increasing emphasis is being placed on elucidating how polysaccharide molecular architecture governs receptor recognition, downstream signaling, and therapeutic efficacy. The present scientometric evidence demonstrates that β-glucans remain the conceptual foundation of *G. frondosa* research because they mechanistically connect polysaccharide chemistry with innate immune activation, cytokine regulation, antitumor activity, and the expanding translational applications of this medicinal mushroom (Kiran et al., 2026).

#### 4.3.2 Antioxidant, anti-inflammatory, and metabolic activities

Scientometric analysis reveals a substantial conceptual shift in *G. frondosa* bioactivity research, moving from a primary focus on immunomodulation to a multidisciplinary framework that includes antioxidant defense, inflammation, metabolic regulation, gut microbiota, and functional food science. This evolution is evidenced by the emergence of themes such as “antioxidant activity,” “oxidative stress,” “hypoglycemic,” “gut microbiota,” “bioactive compounds,” and “structural characterization” in keyword overlays, thematic evolution, and multidimensional scaling analyses. Contemporary research increasingly emphasizes the pleiotropic biological activities of *G. frondosa* rather than solely its immunomodulatory properties. This thematic diversification is supported by the identification of a complex array of bioactive constituents, including β-glucans, heteropolysaccharides, phenolic compounds, ergothioneine, proteins, and other secondary metabolites (Wu et al., 2021). These compounds exhibit complementary and often synergistic effects across multiple physiological systems (Itrat et al., 2025). Among these, antioxidant effects represent one of the most rapidly expanding research areas, as oxidative stress is implicated in the pathogenesis of cancer, diabetes, cardiovascular disease, neurodegeneration, and aging. Experimental evidence indicates that *G. frondosa* polysaccharides and phenolic-rich extracts scavenge reactive oxygen species (ROS), inhibit lipid peroxidation, and enhance endogenous antioxidant enzymes, including superoxide dismutase, catalase, and glutathione peroxidase (Yeh et al., 2011). Furthermore, these bioactive compounds activate the nuclear factor erythroid 2-related factor 2 (Nrf2)/heme oxygenase-1 (HO-1) signaling pathway, restoring redox homeostasis and protecting tissues from oxidative injury (Liu et al., 2022). Closely related to these antioxidant properties are potent anti-inflammatory mechanisms that extend the therapeutic potential of *G. frondosa* beyond traditional immune activation. β-Glucans and other mushroom-derived bioactive compounds suppress excessive activation of nuclear factor-κB (NF-κB), mitogen-activated protein kinase (MAPK), and inflammasome-associated pathways, while regulating the production of inflammatory mediators such as TNF-α, IL-1β, IL-6, nitric oxide, and cyclooxygenase-2 (COX-2) (Xie et al., 2022). These mechanisms connect oxidative stress reduction with immune homeostasis and clarify why inflammation has become a central pathophysiological bridge in current research themes. Scientometric analyses also indicate a growing focus on metabolic health, as evidenced by the recent prominence of “hypoglycemic,” “gut microbiota,” and “functional food” as major research topics. Experimental studies show that *G. frondosa* polysaccharides improve glucose homeostasis by enhancing insulin sensitivity, activating AMP-activated protein kinase (AMPK), suppressing hepatic gluconeogenesis, preserving pancreatic β-cell function, and remodeling gut microbial communities that produce beneficial short-chain fatty acids involved in metabolic regulation (Pan et al., 2020). The integration of gut microbiota into current research reflects a transition from reductionist pharmacological approaches to systems-level understanding of host–microbe interactions, thereby expanding the therapeutic relevance of *G. frondosa* to metabolic syndrome, obesity, and chronic inflammatory diseases. These advances have established *G. frondosa* as a promising nutraceutical and functional food, with diverse bioactive compounds acting as multi-target modulators of oxidative stress, inflammation, immune responses, metabolic homeostasis, and gut microbial ecology (Wu et al., 2021). This progression from single-pathway immunomodulation to integrated systems biology accounts for the thematic diversification observed in the present scientometric analysis and underscores the increasing significance of *G. frondosa* in precision nutrition, healthy aging, and preventive medicine.

#### 4.3.3 Molecular mechanisms underlying bioactivity

The scientometric analyses indicate that the therapeutic potential of *G. frondosa* is increasingly interpreted through integrated molecular mechanisms rather than isolated bioactivities, as reflected by the strong conceptual association of apoptosis, cytokines, oxidative stress, β-glucan, Dectin-1, structural characterization, and immunomodulation across the keyword co-occurrence network, thematic evolution, strategic thematic map, and multidimensional scaling analyses. This trend suggests that recent research has shifted from demonstrating biological efficacy to elucidating the molecular pathways underlying its pharmacological effects. Apoptosis remains a central anticancer mechanism, with *G. frondosa* polysaccharides activating the intrinsic mitochondrial pathway through *BAX* upregulation, Bcl-2 suppression, cytochrome *c* release, mitochondrial membrane depolarization, and caspase-9/-3 activation, culminating in programmed cancer cell death (Zhang et al., 2017). Similarly, β-glucans acts as potent immunomodulatory agent by engaging key pattern-recognition receptors, particularly Dectin-1, complement receptor 3, and Toll-like receptors, thereby activating Syk/CARD9, NF-κB, and MAPK signaling pathways that regulate cytokine production and enhance macrophage, dendritic cell, natural killer cell, and T-cell responses (Ahmad et al., 2024). Moreover, *G. frondosa* polysaccharides have a cryoprotective mechanism by regulating the redox homeostasis and inflammatory response. These bioactive compounds stimulate the Nrf2/HO-1 antioxidant axis while simultaneously inhibiting ROS production and NF-κB-driven inflammatory responses thereby mitigating oxidative damage associated with cancer and other chronic diseases (Xu et al., 2023). Moreover, the emergence of “structural characterization” as a prominent research theme highlights the significance of structure–activity relationships, with molecular weight, β-(1→3)/(1→6)-glucan branching, triple-helical conformation, and polysaccharide architecture governing receptor binding, signaling efficiency, and biological potency (Zhao et al., 2025). The experimental literature collectively identifies interconnected mechanisms, including apoptosis, immune modulation, and redox homeostasis, that may contribute to the reported bioactivities of *G. frondosa* polysaccharides. However, the prominence of these mechanisms in the current scientometric analysis indicates research focus rather than established therapeutic efficacy. Additional clinical validation is necessary to assess the translational relevance of these mechanisms.

### 4.4 Translational barriers to clinical development of *Grifola frondosa*

#### 4.4.1 Predominance of preclinical evidence and limited human validation

Despite more than three decades of research demonstrating the diverse pharmacological activities of *G. frondos*a, the overwhelming majority of evidence remains confined to *in vitro* experiments and animal models. Antitumor, immunomodulatory, antidiabetic, antioxidant, antiviral, and metabolic benefits have been repeatedly reported for its polysaccharides, particularly the D-fraction and other β-glucan-rich preparations (He et al., 2017; Konno, 2009, 2014; Wu et al., 2021). Although these findings establish strong biological plausibility, relatively few well-designed human intervention studies have validated these effects under clinically relevant conditions. A meta-analysis of 24 animal studies further confirmed significant reductions in tumor growth and improvements in immune parameters following *G. frondosa* polysaccharide administration, yet the authors emphasized that the included studies exhibited substantial risks of selection, detection, and reporting bias, thereby limiting confidence in translating these findings directly into clinical practice (Zhao et al., 2021). More broadly, successful translation from promising preclinical discoveries to effective clinical therapies remains exceptionally uncommon across biomedical research, with only an estimated 5–10% of promising preclinical interventions ultimately progressing to positive Phase II clinical trials (Lalu et al., 2020). Although *G. frondosa* has been the subject of extensive preclinical research, there is currently insufficient clinical validation to justify its widespread therapeutic use.

#### 4.4.2 Variability and lack of standardization of bioactive preparations

Another major obstacle to clinical translation is the considerable heterogeneity of *G. frondos*a bioactive preparations. The mushroom contains structurally diverse polysaccharides that differ in β-glucan backbone architecture, branching patterns, molecular weight, monosaccharide composition, protein conjugation, and extraction source (fruiting body, mycelium, or fermentation broth), while additional variability arises from hot-water, alkaline, acidic, microwave, and fermentation-based extraction methods (He et al., 2019; Wu et al., 2021). These methodological differences generate preparations with distinct physicochemical characteristics and biological activities, making direct comparison among studies difficult and reducing reproducibility across laboratories. Although D-fraction and SX-fraction have become relatively well-characterized formulations, many experimental studies continue to investigate heterogeneous extracts with limited chemical characterization, hindering consistent dose standardization and quality control (Konno, 2014). Similar translational challenges have been recognized for other botanical therapeutics, where inadequate raw material standardization, manufacturing consistency, and regulatory compliance substantially delay clinical development (Yadav et al., 2026). Establishing standardized extraction procedures, validated analytical fingerprints, and reproducible manufacturing protocols therefore remains essential before *G. frondos*a preparations can be reliably evaluated in multicenter clinical trials.

#### 4.4.3 Incomplete understanding of pharmacokinetics, bioavailability, and structure-activity relationships

Although β-glucans are recognized as the principal bioactive constituents of *G. frondos*a, important uncertainties remain regarding their absorption, distribution, metabolism, excretion, and structure-dependent biological activity. Numerous polysaccharides possessing different molecular weights, branching structures, and monosaccharide compositions have demonstrated biological activity, yet their comparative pharmacokinetic behavior and mechanisms governing clinical efficacy remain poorly defined (He et al., 2017; Wu et al., 2021). More broadly, mushroom-derived bioactives frequently exhibit poor oral bioavailability and rapid metabolism, limiting systemic exposure despite strong pharmacological activity in experimental models (Inamdar et al., 2025). Similar pharmacokinetic barriers have been identified across natural product research, where low aqueous solubility, limited target specificity, and rapid metabolic clearance substantially restrict therapeutic translation (Yadav et al., 2026). Consequently, the absence of comprehensive pharmacokinetic studies and validated structure–activity relationships limits rational dose optimization, formulation development, and prediction of clinical responses for *G. frondos*a therapeutics.

#### 4.4.4 Limited mechanistic validation in human biology

Most mechanistic studies of *G. frondosa* have been conducted using simplified experimental systems that cannot fully replicate the complexity of human physiology. Although numerous investigations have demonstrated immunomodulatory signaling, cytokine regulation, and antitumor activity, relatively few studies have verified whether these molecular mechanisms operate similarly in patients (Konno, 2009; He et al., 2019). More generally, translational research frequently relies on experimental models that inadequately reflect human genetics, disease heterogeneity, and pathophysiology, resulting in therapeutic concepts that perform well experimentally but fail during clinical evaluation (Marincola, 2011). Emerging perspectives further emphasize that conventional *in vitro* models often overlook biological variability, interconnected signaling networks, temporal regulation, and patient-specific responses, thereby reducing their predictive value for human outcomes (Madorran, 2026). Consequently, although mechanistic evidence supporting *G. frondos*a bioactivity continues to expand, stronger validation using human-relevant experimental models, predictive biomarkers, and clinically meaningful mechanistic endpoints remains necessary to improve translational confidence.

#### 4.4.5 Regulatory, manufacturing, and clinical development challenges

Successful clinical translation requires standardized manufacturing, regulatory approval, long-term safety assessment, and scalable production. Encouragingly, the D-fraction has demonstrated a favorable safety profile, receiving exemption from Phase I toxicology testing by the United States Food and Drug Administration and approval for an Investigational New Drug application for a Phase II pilot study involving patients with advanced cancers (Konno, 2014). Likewise, a *G. frondos*a polysaccharide-based drug was approved in China in 2010 as an adjunctive cancer therapy (He et al., 2019). Nevertheless, broader international clinical implementation remains constrained by regulatory requirements for manufacturing consistency, quality assurance, pharmacovigilance, and long-term clinical evidence. Experiences from mushroom-derived therapeutics and other natural products consistently indicate that good manufacturing practice compliance, scalability, safety documentation, and harmonized regulatory standards remain major prerequisites for commercialization (Inamdar et al., 2025; Yadav et al., 2026). Similar barriers have also been identified for biologically active therapeutics more broadly, where production complexity, delivery optimization, and manufacturing costs continue to impede widespread clinical adoption despite promising biological efficacy.

#### 4.4.6 Weak integration between preclinical and clinical research

A persistent barrier underlying many of the aforementioned challenges is the insufficient integration between laboratory discoveries and clinical investigation. Translational research has historically prioritized bench-to-bedside progression without adequately incorporating clinical feedback to refine experimental models, identify predictive biomarkers, or explain discrepancies between anticipated and observed therapeutic outcomes (Marincola, 2011). Consequently, many promising bioactive compounds continue to be evaluated using experimental paradigms that fail to capture clinically relevant disease complexity. Lalu et al. (2020) similarly emphasized that deficiencies in preclinical study design, reporting quality, and methodological rigor substantially reduce the likelihood of successful clinical translation. For *G. frondos*a, strengthening multidisciplinary collaboration among mycologists, pharmacologists, clinicians, pharmaceutical scientists, and regulatory agencies will be essential to establish standardized protocols, clinically meaningful endpoints, and evidence-based translational frameworks capable of advancing promising mushroom bioactives from experimental research to validated therapeutic applications.

### 4.5 Emerging research frontiers and future research priorities

Despite the considerable pharmacological potential of *G. frondosa*, its effective translation into standardized therapeutic and functional food products necessitates coordinated progress in fungal biology, biotechnology, pharmacology, food science, and clinical medicine. Establishing standardized platforms for cultivation, extraction, and quality control is a primary research objective to ensure the production of bioactive preparations with consistent chemical composition and biological efficacy. Extraction methods, purification procedures, and thermal processing significantly affect polysaccharide molecular weight, branching architecture, and antitumor activity. Therefore, future research should combine optimized cultivation protocols with validated analytical fingerprinting to produce reproducible products suitable for regulatory approval (Li and Liu, 2020; Sun et al., 2023; Ren et al., 2025; Zeb et al., 2025). Identifying optimal liquid culture conditions and developmental regulators, such as heat shock proteins and cyclic adenosine monophosphate signaling pathways, offers further opportunities to enhance industrial-scale production and reduce batch-to-batch variability (Ren et al., 2025). These manufacturing improvements are critical for advancing both pharmaceutical development and the commercial production of functional foods (Camilleri et al., 2024; Sadowska et al., 2026).

Translational research should progress beyond descriptive bioactivity studies and focus on mechanistic and precision-guided therapeutic development. There is a need to prioritize the elucidation of structure-activity relationships, receptor interactions, pharmacokinetics, metabolism, bioavailability, and potential drug interactions using quantitative experimental methodologies (Camilleri et al., 2024; Wu et al., 2021). Recent findings underscore the significance of this approach. For instance, the homogeneous β-glucan GFPS acts as a dectin-1-mediated carrier for antisense oligonucleotide delivery, demonstrating the potential of *G. frondosa* polysaccharides as biomaterials for targeted nucleic acid therapeutics rather than solely as immunostimulatory agents (Cui et al., 2020). Similarly, the structurally distinct α-glucan GFI-21α has shown strong *in vivo* antitumor efficacy by enhancing intratumoral CD8^+^ T-cell activation and interferon-γ-associated cytotoxicity, indicating opportunities to develop next-generation mushroom-derived cancer immunotherapeutics with defined molecular targets (Tian et al., 2026). Ongoing integration of structural chemistry, glycobiology, immunology, and drug delivery technologies will accelerate the identification of clinically relevant lead compounds.

A critical translational priority is the generation of high-quality clinical evidence to bridge laboratory discoveries with medical practice. Although numerous *in vitro* and animal studies exist, randomized controlled trials assessing standardized *G. frondosa* preparations are limited, which restricts evidence-based recommendations for oncology, metabolic disorders, inflammatory diseases, and immune-mediated conditions (Rossi et al., 2018; Zeb et al., 2025). Future research should include multicenter clinical trials that evaluate long-term efficacy, safety, quality of life, immune biomarkers, pharmacodynamic responses, and compatibility with conventional therapies, especially in cancer management (Rossi et al., 2018). Concurrently, the integration of *G. frondosa* into scientifically validated functional foods and nutraceuticals will require quantitative assessments of bioavailability, efficacy, consumer safety, and regulatory compliance to facilitate commercialization (Camilleri et al., 2024; Sadowska et al., 2026). Expanding therapeutic applications beyond oncology, such as for inflammatory skin diseases mediated by mitogen-activated protein kinase pathway inhibition, further broadens the clinical landscape. This expansion underscores the necessity for interdisciplinary collaboration among mycologists, pharmacologists, immunologists, clinicians, food technologists, and industrial partners to fully realize the therapeutic and commercial potential of *G. frondosa* (Choi and Choi, 2023).

## 5 Limitations of the study

While this study offers a comprehensive scientometric overview of global bioactivity research on *Grifola frondosa*, several limitations should be considered. First, the analysis relied exclusively on Scopus, excluding records indexed only in Web of Science, PubMed, regional databases, books, patents, conference proceedings, and gray literature. This may have resulted in uneven geographical and disciplinary coverage. Second, the search was restricted to English-language journal articles published through 2025, potentially omitting relevant studies published in other languages, particularly from countries with established traditions in medicinal mushroom research such as China, Japan, and South Korea. This language restriction has direct implications for the geographical findings: country-level publication counts and rankings should be interpreted as measures of visibility within the English-language Scopus-indexed literature rather than as complete estimates of national research activity. Countries with stronger traditions of publishing in local-language or non-Scopus-indexed journals may therefore appear less productive than they actually are, while countries with greater representation in international English-language journals may be comparatively more visible. Accordingly, the observed geographical concentration in East Asia reflects the structure of the indexed dataset and should not be regarded as an exact representation of the global distribution of *G. frondosa* research. Third, bibliometric indicators such as publication counts, citation metrics, and collaboration networks describe scientific activity but do not evaluate methodological quality, experimental rigor, or clinical significance. Citation-based measures are also affected by publication age and citation-window effects, potentially underestimating the influence of recent articles. Finally, despite manual data cleaning and keyword harmonization, residual variations in author names, institutional affiliations, and indexing practices may have introduced minor inaccuracies. Future studies should consider multilingual searches and the integration of Scopus with complementary international and regional databases to obtain a more inclusive assessment of geographical participation and research development.

## 6 Conclusion

This scientometric analysis demonstrates that bioactivity research on *Grifola frondosa* has evolved from an early emphasis on immunomodulatory polysaccharides into an increasingly multidisciplinary and internationally connected field encompassing pharmacology, oncology, immunology, biotechnology, food science, metabolomics, and nutraceutical development. The sustained growth in publications, expansion of international collaborations, and diversification of research themes indicate increasing scientific attention to *G. frondosa* as a source of bioactive compounds with potential applications in health promotion and disease prevention. Although bioactive polysaccharides, particularly β-glucans, remain prominent research themes, emerging work on structural characterization, gut microbiota modulation, fermentation technologies, oxidative stress, metabolic health, and functional food development indicates a broadening of the research landscape toward mechanistic and translational investigations. Within the English-language Scopus-indexed literature analyzed, China and Japan remain major centers of research productivity and collaboration, while increasing participation from other countries reflects the expanding international scope of the field. Thematic patterns further suggest opportunities for greater integration of food science, molecular and systems biology, microbiome research, bioengineering, nutrition, and clinical sciences. Importantly, translating these scientific advances into greater public interest and dietary adoption will require positioning *G. frondosa* as an accessible, scientifically supported, and culturally acceptable functional food rather than solely as a medicinal product. This may be facilitated through a translational pathway encompassing bioactive-compound characterization, standardized cultivation and processing, development of palatable and convenient food formulations, rigorous preclinical and clinical evaluation, substantiation of health claims, and effective consumer education. Incorporating *G. frondosa* into familiar food formats, such as soups, beverages, powders, snacks, seasonings, and fermented products, may facilitate routine consumption, while transparent labeling, standardized bioactive content, quality and safety assurance, appropriate regulatory oversight, and clear communication of scientifically supported benefits could strengthen consumer confidence. Greater collaboration among researchers, food manufacturers, nutrition and healthcare professionals, policymakers, and community stakeholders may help bridge scientific research and consumer acceptance, while continued mechanistic and clinical investigations will be necessary to strengthen the evidence base supporting the integration of *G. frondosa* into preventive nutrition and health-maintaining dietary practices.

## Data Availability

The original contributions presented in the study are included in the article/supplementary material. Further inquiries can be directed to the corresponding author.
